# Analytic description of nanowires II: morphing of regular cross sections for zincblende- and diamond-structures to match arbitrary shapes

**DOI:** 10.1107/S2052520622004942

**Published:** 2022-07-15

**Authors:** Dirk König, Sean C. Smith

**Affiliations:** a Integrated Materials Design Lab (IMDL), Research School of Physics and Engineering, The Australian National University, ACT 2601, Australia; b Institute of Semiconductor Electronics (IHT), RWTH Aachen University, 52074 Aachen, Germany; c Integrated Materials Design Centre (IMDC), University of New South Wales, NSW 2052, Australia; d Department of Applied Mathematics, Research School of Physics and Engineering, The Australian National University, ACT 2601, Australia; Polish Academy of Sciences, Poland

**Keywords:** nanowire cross section, metrology, morphing, zincblende, diamond

## Abstract

Setting out from our recent publication [König & Smith (2021 ▸). *Acta Cryst.* B**77**, 861], we extend our analytic description of the regular cross sections of zincblende- and diamond-structure nanowires (NWires) by introducing cross section morphing to arbitrary convex shapes featuring linear interfaces as encountered in experiment. To this end, we provide add-on terms to the existing number series with their respective running indices for zinc-blende- (zb-) and diamond-structure NWire cross sections. Such add-on terms to all variables yield the required flexibility for cross section morphing, with main variables presented by the number of NWire atoms *N*_Wire_(*d*_Wire_[*i*]), bonds between NWire atoms *N*_bnd_(*d*_Wire_[*i*]) and interface bonds *N*_IF_(*d*_Wire_[*i*]). Other basic geometric variables, such as the specific length of interface facets, as well as widths, heights and total area of the cross section, are given as well. The cross sections refer to the six high-symmetry zb NWires with low-index faceting frequently occurring in the bottom-up and top-down approaches of NWire processing. The fundamental insights into NWire structures revealed here offer a universal gauge and thus enable major advancements in data interpretation and the understanding of all zb- and diamond-structure-based NWires with arbitrary convex cross sections. We corroborate this statement with an exact description of irregular Si NWire cross sections and irregular InGaAs/GaAs core-shell NWire cross sections, where a radially changing unit-cell parameter can be included.

## Abstract 2

Setting out from König & Smith (2021), we present an analytic morphing of zincblende- and diamond-structure nanowire (NWire) cross sections to arbitrary convex shapes as encountered in experiment. We predict the NWire atoms, bonds between these atoms and NWire interface bonds, plus characteristic lengths and area of the NWire cross sections. Cross section areas, ratios of internal bonds per NWire atom enable an interpretation of *any* spectroscopic NWire data. Our algorithms include a radial dependence of the NWire unit-cell parameter and can be applied to multi-core-shell NWires where NWire layers can be morphed independently from each other.

## 1. Introduction

**Figure 1:**
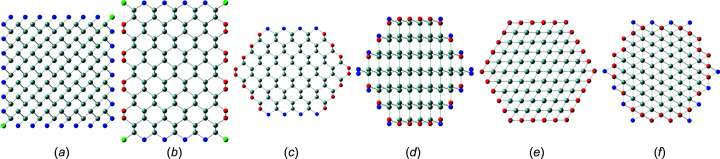
Examples of regular cross sections of zb-NWires as described analytically in König & Smith (2019 ▸, 2021 ▸). (*a*) Square cross section with a [001] growth vector and {001} inter­faces, (*b*) rectangular cross section with a [110] growth vector and two {111} plus two {001} inter­faces, (*c*) hexa­gonal cross section with a [110] growth vector and four {110} plus two {111} inter­faces, (*d*) hexa­gonal cross section with a [11 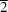 ] growth vector and two {111} plus four {1 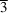 1} inter­faces, (*e*) hexa­gonal cross section with a [111] growth vector and six {110} inter­faces, and (*f*) hexa­gonal cross section with a [111] growth vector and six {11 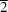 } inter­faces. Colour code of the inter­face atoms: red has one inter­face bond each, blue has two inter­face bonds each and green has three inter­face bonds each.

In recent publications, we derived (König & Smith, 2019 ▸) and improved (König & Smith, 2021 ▸) the analytical description of six regular zb-NWire cross sections relevant to experiment (Weber & Mikolajick, 2017 ▸); see Fig. 1 ▸. To this end, we described the number of atoms, the number of bonds between such atoms and the number of interface bonds for an NWire slab with a thickness of the periodic unit cell (UC) along its growth axis with its interface length, height, width and NWire cross section area. An analytical structural description of the NWire cross section down to the individual bond and atom is a powerful tool for interpreting or predicting (König *et al.*, 2021 ▸) any experimental data as a function of NWire cross section size, shape and orientation of its growth axis and interfaces. Here, we aim to extend this analytic description for zb- and diamond-structure NWires to arbitrarily convex cross sections featuring linear interfaces, thereby allowing one to fit the analytics of such cross sections to any irregular convex shape encountered in experiment.

Section 2 provides the necessary background information on the nomenclature on how to interpret the cross section images per NWire type, and a brief assignment of primary and secondary parameters to structure-driven phenomena. Section 3 contains the number series of all six different NWire cross sections, as shown in Fig. 1 ▸ for uniaxial morphing (*C*_2_ symmetry). In Section 4, we introduce triaxial morphing to all four hexagonal cross sections (*C*_3_ symmetry) with three independent run indices, allowing for a vast range of cross section shapes. Combining the morphing algorithm in both sections, virtually any crystalline zb-NWire with convex cross section geometry can be described. In Section 5, we show examples of applying the number series and derived secondary parameters to experimental data from the literature for each, irregular Si and core-shell III–V NWires. Appendices *A* [App appa], *B* [App appb] and *C* [App appc] derive characteristic lengths and areas for cross sections with [110], 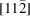 and [111] growth vectors, respectively. As this work builds upon our previous publications, we refer the reader to König (2016 ▸) and König & Smith (2021 ▸) for the background information on chosen cross sections, interface energetics, bond densities and further details regarding the basics of associated analytic number theory.

## 2. General remarks on analytic number series, structural boundary conditions and nomenclature

Table 1 ▸ lists the primary and secondary parameters calculated by number series.

All parameters are calculated over an NWire slab presenting the thickness of the UC *a*_uc_ in the growth direction as per König & Smith (2021 ▸) to achieve periodicity (Table 2 ▸). In addition, Table 2 ▸ lists the amount of atoms and bonds per column (*i.e.* per atom or bond visible) as a function of NWire axis orientation for a top view onto the cross section, thereby allowing atoms and bonds to be counted. Respective images are provided for all NWire cross section types presented here.

The periodicity in the growth direction and the assumption that the length of the NWire 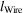 exceeds its diameter 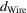 allows for a highly accurate description of parameters, though, as per mathematical definition, they are correct only for 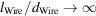 .

On a par with König & Smith (2021 ▸), the indexing of NWire cross section type is given as a superscript with its shape and growth direction; see Table 3 ▸.

With 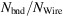 , we obtain a gauge for the response to internal stress, *e.g.* by dopant species. The ability of embedding materials or ligands to exert stress (Schuppler *et al.*, 1994 ▸; Boyd & Wilson, 1987 ▸) onto NWires or *vice versa* can be described with 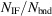 . The impact of a highly polar surface termination on the zb-NWire electronic structure observed as interface-related electronic phenomena (Zahn *et al.*, 1992 ▸; Campbell *et al.*, 1996 ▸; He *et al.*, 2009 ▸; König *et al.*, 2014 ▸, 2018 ▸, 2019 ▸, 2021 ▸) is assessed by the ratio 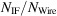 . The ratio 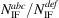 can be useful for detecting facet-specific interface defects. For Si, interface-specific dangling bond (DB) defects exist, namely, the P 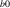 centre at {001} interfaces and the P 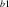 centre at {111} interfaces (Helms & Poindexter, 1994 ▸; Keunen *et al.*, 2011 ▸). These DB defects occur in a ratio which reflects 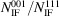 and can be detected by electron paramagnetic resonance (EPR) (Stesmans *et al.*, 2008 ▸). For Si-NWires, the ratio 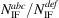 is therefore a valuable tool for identifying cross sections of the NWires treated in Sections 3.2, 4.1 and 4.2. We illustrate the results on tetrahedral C, Si and Ge NWires (all diamond structure). NWire atoms without interface bonds are shown in grey. Atoms with interface bonds are colour-coded: species with one/two/three interface bonds are red/blue/green; see Fig. 1 ▸ for an example. The analytical number series introduced below also hold for zb-NWires due to straightforward symmetry arguments (König & Smith, 2021 ▸). Material properties resulting from differences in the base cell – *A*–*B* for zb-structures *versus* *A*–*A* for diamond structures – are not considered here. This constraint has no impact on the applicability of the analytics of our work, unless the atomic sequence mentioned above is of primary interest when comparing two solids.

**Figure 2:**
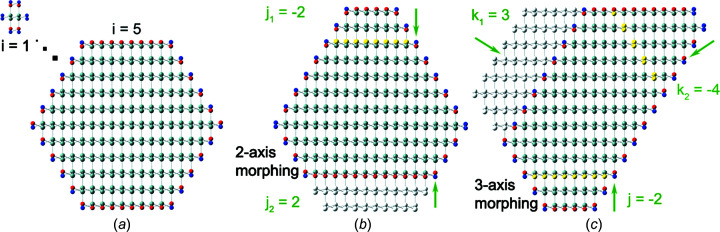
Example of the axial-symmetric morphing shown with the members of the *even* series of regular hexa­gonal zb-NWire cross sections with a [11 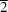 ] growth vector and two {111} inter­faces at the top and bottom, plus four { 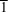 31} side inter­faces; see König & Smith (2021 ▸) and Sections 3.4 and 4.2 for details. (*a*) Nominal shapes for run indices *i* = 1 (*X*_16_), growing to *i* = 5 (*X*_320_). From the nominal shape and any value of *i* (here for *i* = 5), *lateral* number series are introduced to morph the nominal cross section. (*b*) Two lateral run indices *j*_1_ and *j*_2_ are introduced to allow for independent morphing in the 〈111〉 direction from the top and bottom inter­faces [(111) and ( 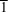 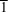 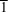 ), respectively], maintaining symmetry along the vertical axis; the nominal cross section occurs for *j*_1_ = *j*_2_ = 0, as shown by white ‘ghost atoms’ for *j* > 0, and by the nominal (111) inter­face illustrated by yellow atoms for *j* < 0. (*c*) Morphing along three directions with run indices *j* = −2 [expansive morphing, shifting ( 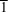 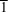 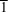 ) inter­face], *k*_1_ = 3 [reductive morphing, shifting (3 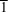 1) inter­face] and *k*_2_ = −4 [expansive morphing, shifting ( 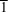 31) inter­face].

The nominal number series describing the high-symmetry NWire cross sections follow a run index *i* which defines the *nominal size* of the cross section. Morphing of cross sections is introduced by a second class of run indices 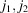 ( 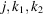 or 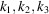 ) for *C*_2_ symmetry uniaxial (*C*_3_ symmetry triaxial) morphing, defining the *shape* – or more precisely, its deviation from the respective high-symmetry cross section. For quandrangle cross sections treated in Sections 3.1 and 3.2, one index *j* is sufficient to describe the symmetry deviations elaborated here, as is straightforward to see by turning cross sections by 90°. For the four remaining more complex hexagonal cross sections, we introduce two running indices 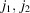 to allow for independent morphing from the top and bottom interfaces. Generally, we have 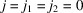 for the nominal shape of the cross section. The morphing indices then span the range of 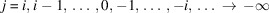 for quadrangle cross sections, and – with one exception (see Section 3.5) – of 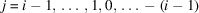 for hexagonal cross sections, the positive limit of the latter occurring due to their interface planes intersecting at finite distance for 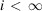 (as opposed to parallel interfaces for quadrangle cross sections). An example of cross section morphing is shown in Fig. 2 ▸. These limits to lateral run indices for hexagonal cross sections are also valid in triaxial morphing with lateral run indices 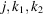 or 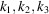 (Sections 4 and 5.1), again with one exception (Sections 4.3 and 5.2).

For the hexagonal cross sections, we originally developed an *even* and an *odd* series to account for minor deviations from the high-symmetry cross sections in experiment (König & Smith, 2021 ▸). The differences between parameters from *odd versus even* series are outrun by far with the modifications due to morphing. As a consequence, we introduce morphing here only to the *even* series of all hexagonal cross sections. While adequate number series modifications can also be derived for the *odd* series of all hexagonal cross sections, their descriptions of NWire cross sections are covered by morphing the *even* series onto experimental data. An example is the cross sections of Si NWires with a [110] growth axis and {001} plus {111} interfaces; see Fig. 1 ▸(*c*) herein and the experimental data published by Yi *et al.* (2011 ▸). This cross section was treated in König & Smith (2019 ▸) with *even* and *odd* cross section calculus. With the morphing introduced in Section 3.3 and, in particular, in Section 4.1, we can simply use the *even* series and morph it exactly onto the experimental image.

## 3. Morphing cross sections along one symmetry axis

Terms describing the high-symmetry cross section (*i.e.* 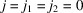 ) are identical with the respective Equations in König & Smith (2021 ▸). Such terms are printed here in grey to distinguish them from terms due to morphing which are printed in black.

### 3.1. Nanowires growing along the [001] direction with square cross section and four {001} interfaces

As mentioned briefly in the *Introduction*, run indices for this cross section are limited to 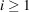 and 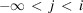 .


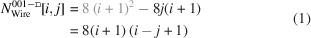





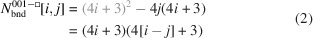




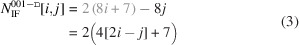




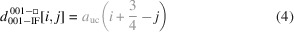

The square shape of the cross section results in 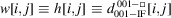 .

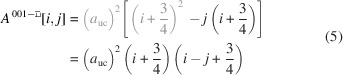

**Figure 3:**
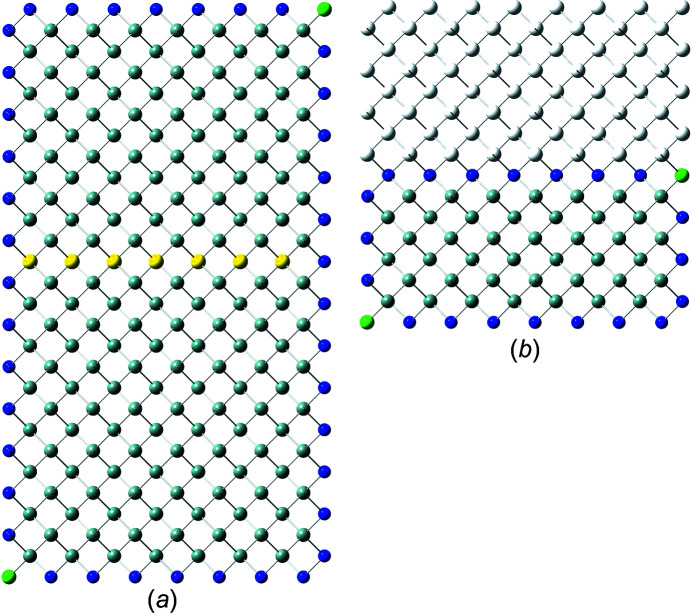
Cross section of the zb-/diamond-structure NWires growing along the [001] axis with a square cross section and four {001} inter­faces for run index *i* = 3 and (*a*) expansive morphing with index *j* = −3, and (*b*) reductive morphing with index *j* = 2. The latter morphing is not considered useful since a 90° rotation yields to expansive morphing at a lower run index *i*, thereby restricting *j* to negative integers (here: *i* = 1 and *j* = −2). Yellow atoms show the outer limit of the nominal cross section (*i* = 3 and *j* = 0) in part (*a*) and white atoms in part (*b*) present ‘ghost atoms’ to fill up the nominal cross section. Inter­ior atoms are grey, atoms with two inter­face bonds are blue and atoms with three inter­face bonds are green.

Fig. 3 ▸ shows morphing examples of the square NWire cross sections with growth along the [001] direction and four {001} interfaces.

### 3.2. Nanowires growing along the [110] direction with a rectangular cross section and two {001} plus two {110} interfaces

As mentioned briefly in the *Introduction*, run indices for this cross section are limited to 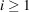 and 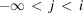 .

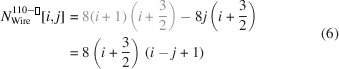




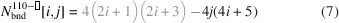




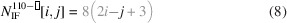

Since we morph the cross section along the {110} interfaces, 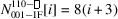 does not change with *j*. However, 

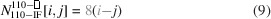

is a function of *j* by which the ratio of interface bonds between facets becomes 

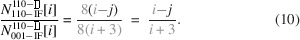

The centre expression shows both number series in their explicit form, while the expression on the right presents the simplified result of their ratio.

As was the case for 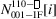 , the length of the {001} interface remains unchanged;

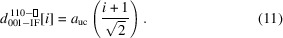

The width of the rectangular cross section follows in a straightforward manner from 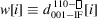 . Morphing has an impact on the length of the {110} interfaces, resulting in 

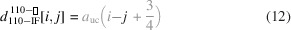

The height of the rectangular cross section follows in a straightforward manner from 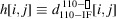 .

The total cross section area is given by 

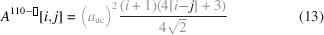

**Figure 4:**
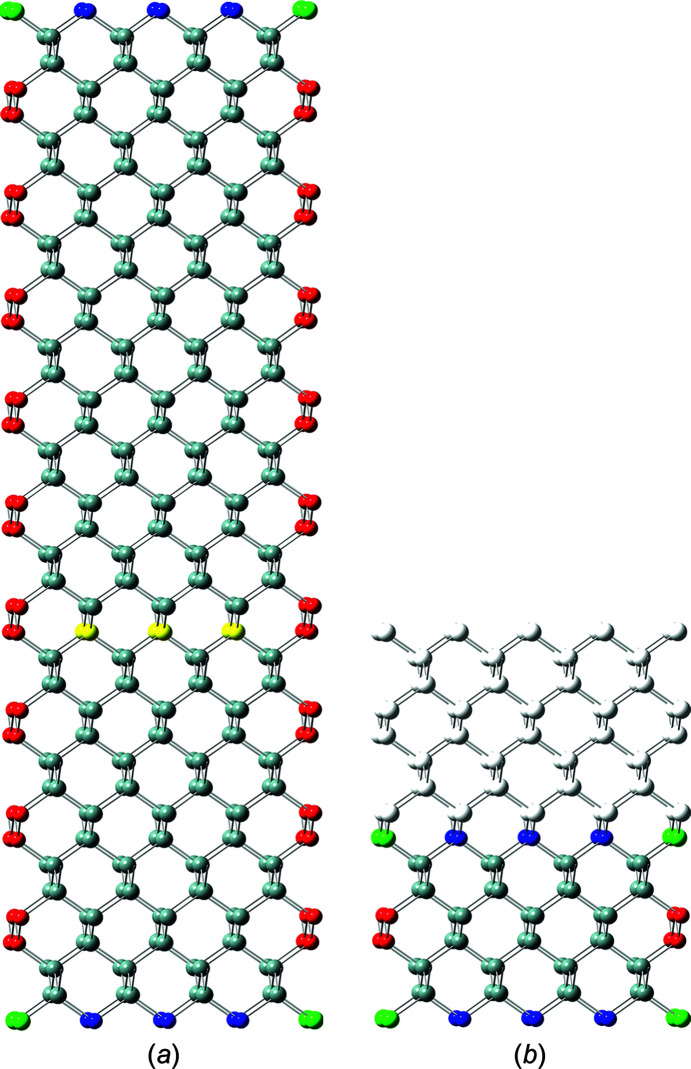
Cross section of the zb-/diamond-structure NWires growing along the [110] axis with a rectangular cross section and two {001} inter­faces at the top and bottom, and two {110} inter­faces at the sides, for run index *i* = 3, showing (*a*) expansive morphing with morphing index *j* = −6 and (*b*) reductive morphing with *j* = 2. Yellow atoms show the outer limit of the nominal cross section (*i* = 3 and *j* = 0) in part (*a*), white atoms in part (*b*) present ‘ghost atoms’ to fill up the nominal cross section. Inter­ior atoms are grey, atoms with one inter­face bond are red, atoms with two inter­face bonds are blue and atoms with three inter­face bonds are green. Due to two different inter­face orientations, *j* > 0 is useful to calculate expansive morphing in the horizontal direction (*i.e.* parallel to the {001} inter­faces) by picking an appropriate *i* to match 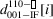 and then reducing the vertical extension by *j* > 0.

The cross section of this NWire type is shown in Fig. 4 ▸.

### 3.3. Nanowires growing along the [110] direction with a hexagonal cross section and four {111} plus two {001} interfaces

The remaining four NWire types to be investigated all have a hexagonal cross section which has a more complex geometry. As mentioned briefly in the *Introduction*, run indices for these cross sections are limited to 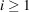 and 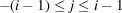 , except for the cross section with an exclusive {110} interface and a [111] growth axis; see Section 3.5.

For the number of atoms forming the NWire cross section, we get 

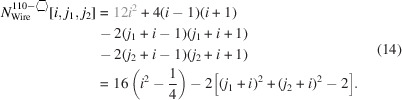

The number of bonds between these atoms is described by 

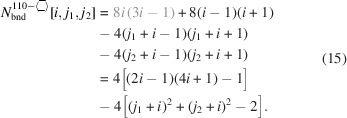

The number of interface bonds over all facets is given by 

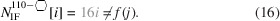

**Figure 5:**
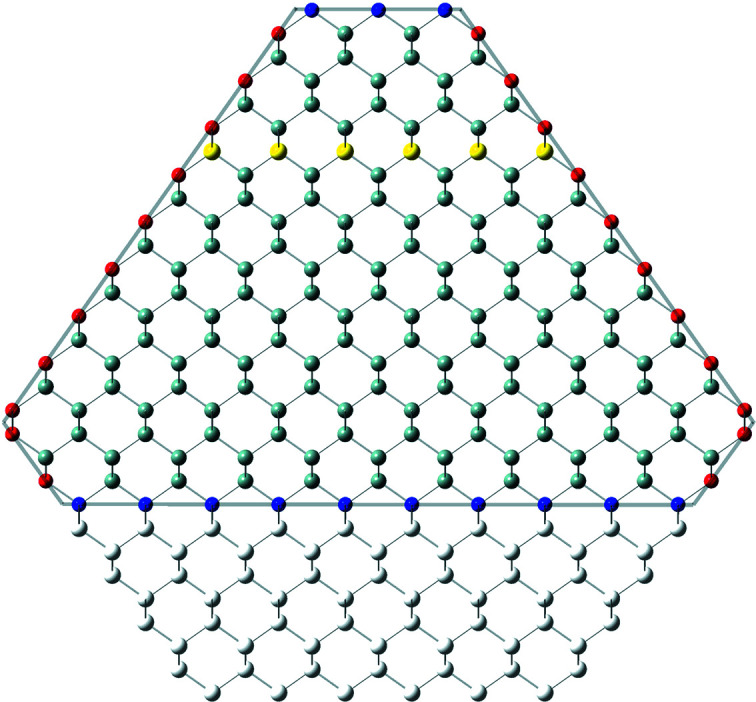
Cross section of the zb-/diamond-structure NWire growing along the [110] axis with a hexa­gonal cross section and four {111} plus two {001} inter­faces for run index *i* = 6, shown with axial-symmetric morphing which is expansive on top with index *j*_1_ = −3, and reductive at the bottom with index *j*_2_ = 4. Translucent lines show the outer limit of the cross section and respective facet lengths. Observe the irregular triangular areas at the apexes of the cross section which are a constant offset to the total cross section area, its width and facet lengths. For atom colours, refer to Fig. 4 ▸. For a detailed geometrical derivation of characteristic lengths and areas, refer to Appendix *A* [App appa].

Since the number of {111} facet bonds being added or removed equals the number of {001} facet bonds being removed or added per change in 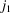 or 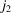 , such contributions cancel each other out; see Equation 17 below. For a graphical verification, we refer the reader to Fig. 5 ▸, or to Fig. 4 in König & Smith (2021 ▸).

The ratio of interface bonds per facet orientation is given by 

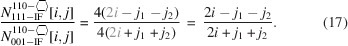

The lengths of the {001} and {111} facets depend only on one lateral run index 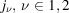 , which affects the respective facet. For the {001} facet, a scaled offset of 1 exists for the two irregular triangular areas in the apexes: 

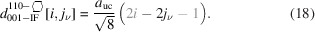

For the {111} facet, a scaled offset of −1/4 exists due to the two irregular triangular areas in the apexes: 

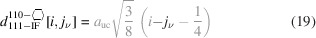

Due to morphing along the vertical symmetry axis of the cross sections, 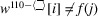 and thus stays unchanged. The scaled offset due to the two isosceles triangles at the side apexes of the cross section is 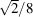 which is added to the nominal increment of 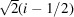 : 

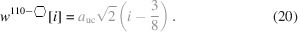

Obviously, the height of the cross section does change with 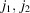 in steps of 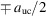 per 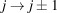 , resulting in 

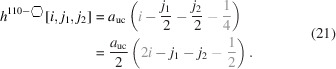

The total cross section area is described by 

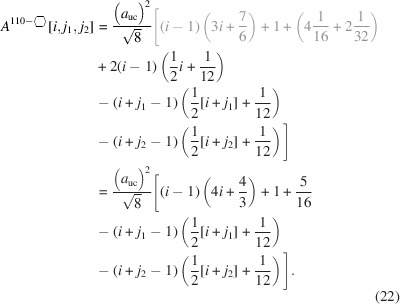

The prefactor 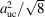 presents the area of one *X*_6_ ring, seen along the 〈110〉 lattice vector, which is straightforward to derive from four such rings filling the zb-UC cross section when cut along the {110} plane, covering an area of 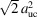 ; see Appendix *A* [App appa]. The offset areas concern the isosceles triangles at the {001} facets with an area of 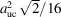 , and the irregular triangles at the {111} facets with 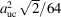 , both of which can be found when considering an *X*_6_ ring seen along the 〈110〉 lattice vector, using geometrical arguments. The total offset area 4/16+2/32 presents the four scalene triangles at the two lower and upper apexes of the cross section, plus the two isosceles triangles occurring at the left- and rightmost apexes of the cross section^1^, see Figs. 5 ▸ and 16, Appendix *A* [App appa] and Fig. 4 in König & Smith (2021 ▸).

### Nanowires growing along the [ 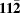 ] direction with hexagonal cross section and four { 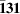 } plus two {111} interfaces

3.4.

For the number of atoms forming the NWire cross section, we get 

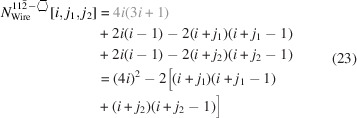

The number of bonds between these atoms is described by 

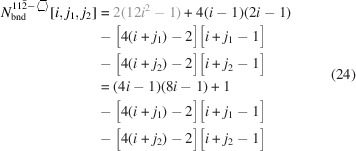

The number of interface bonds over all facets is given by 

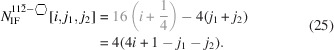

The assignment of interface bonds to the {111} and {1 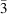 1} facets is shown in Fig. 10(*b*). With this assignment of interface atoms to {111} and {1 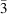 1} facets, we obtain the ratio of interface bonds per facet orientation as 

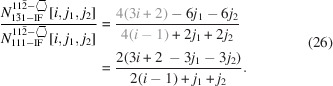

Equation 26 shows the explicit number series in the top line, while the bottom line is the compacted version for the ratio of interface bonds. The following facet lengths depend only on one lateral run index 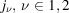 , which is assigned to the facet of interest: 

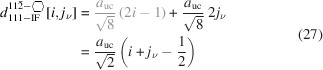

and 

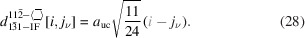

For the 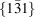 facets, the smallest unit is the diagonal of the congruent rectangular areas constituting the cross section area. These rectangles have a horizontal scaled length of 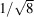 (see Equation 29) and a vertical scaled length of 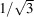 (see Equation 30), yielding 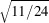 for the scaled diagonal of the rectangle. Due to morphing along the vertical symmetry axis of the cross sections, 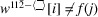 and thus stays unchanged: 

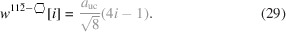

The height of the cross section obviously changes with morphing, following 

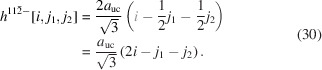

The length 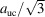 presents a third of the diagonal connecting two opposite corners in 〈111〉 direction through the zb-UC, whereby the (111) vector is orthonormal to the 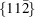 plane; 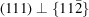 . This length is equivalent to the longer side of the rectangle which presents the unit area of NWires growing along the 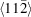 vector class, accounting for the increment in 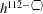 in Equation 30; see Fig. 2 ▸. The total cross section area is described by 

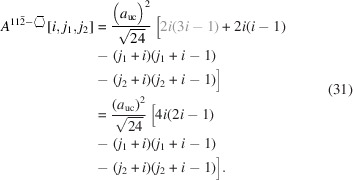

The scaled coefficient of 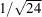 describes the rectangular unit area of the cross section as discussed above, following from 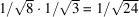 . Facets cut the outermost rectangles along their diagonal, rendering their triangular area 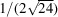 . For an illustration of the morphed hexagonal cross section with {111} top and bottom interfaces plus {11 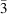 } side interfaces, refer to Figs. 2 ▸ and 10. For a detailed geometrical derivation of characteristic lengths and areas, see Appendix *B* [App appb].

### 3.5. Nanowires growing along the [111] direction with a hexagonal cross section and six {110} interfaces

The smoother geometry of this cross section allows us to use lateral run indices of 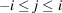 for morphing.

For the number of atoms forming the NWire cross section, we get 

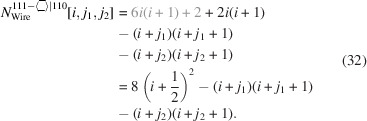

The number of bonds between these atoms is described by 

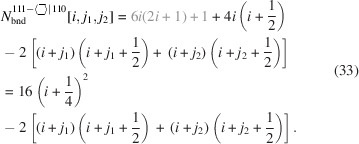

The number of interface bonds over all facets is given by 

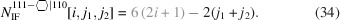

The facet lengths depending on the respective 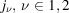 , are 

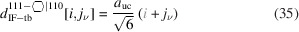

for the top and bottom facets, and 

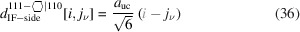

for the side facets. The scaled coefficient 1/ 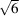 refers to the side length of the equilateral triangles which form the unit area unit on a {111} plane defining the cross section. This coefficient follows from a {111} plane cut through the zb-UC along its corner points, resulting in an equilateral triangle of scaled side length 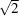 , containing an area equivalent to 12 small equilateral triangles (6 equilateral + 6 isosceles with same area = 12) with a scaled side length of 1/ 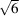 .

The width of the cross section is not a function of *j* and thus stays unchanged: 

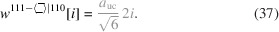

The height of the cross section depends on 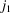 and 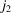 as it is parallel to the symmetry axis along which axial-symmetric morphing occurs: 

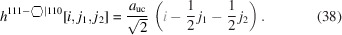

The total cross section area is described by 

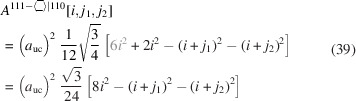

**Figure 6:**
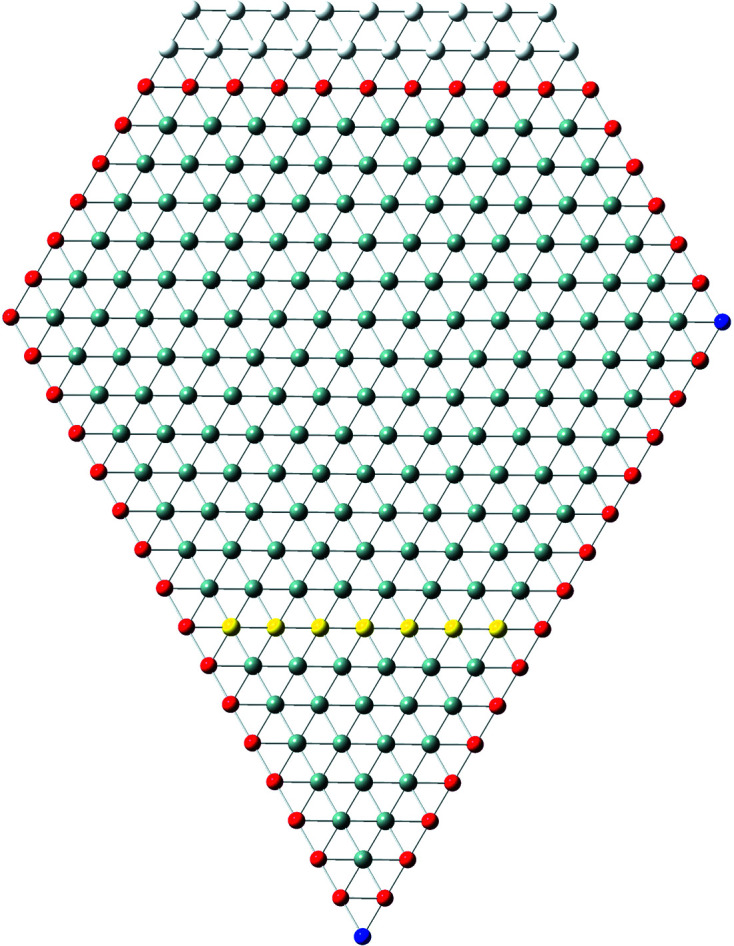
Axial-symmetric morphing of the zb-/diamond-structure NWires growing along the [111] axis with a hexa­gonal cross section and six {110} inter­faces. The nominal cross section for *i* = 8 is shown by white ‘ghost atoms’ at the top inter­face and by the yellow atoms in the lower half of the cross section. Reductive morphing with *j*_1_ = 2 was applied to the top inter­face, while morphing to maximum expansion with *j*_2_ = −*i* = −8 was applied to the bottom inter­face. For atom colours, refer to Fig. 4 ▸. For a detailed geometrical derivation of characteristic lengths and areas, refer to Appendix *C* [App appc].

The scaled coefficient of 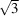 /24 describes the area per equilateral triangle as the unit area of the cross section and follows directly from our discussion of facet lengths above. Fig. 6 ▸ shows the cross section of this NWire type.

### Nanowires growing along the [111] direction with a hexagonal cross section and six { 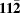 } interfaces

3.6.

This cross section returns to the nominal limitation of lateral (morphing) run indices, *i.e.* 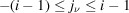 with 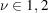 . For the number of atoms forming the NWire cross section, we get 

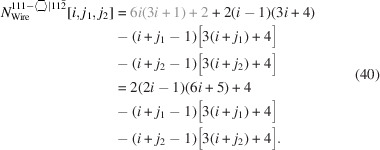

The number of bonds between these atoms is described by 

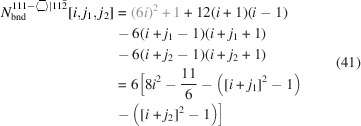

The number of interface bonds over all facets is given by 

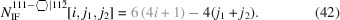

The facet lengths of top and bottom interfaces depend on the respective 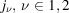 : 

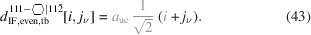

The facet length of side interfaces is 

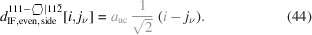

Since the width of this hexagonal cross section is not a function of 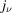 , it remains unchanged: 

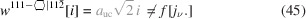

For the height of the cross section, we get 

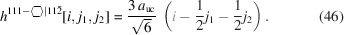

The cross section plane has the same orientation as in Section 3.5, with the facet orientation rotated by 60° ({110} 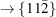 ). This rotation swaps the scaled coefficients of facet lengths and cross section width on one side and cross section height on the other side when compared to Section 3.5 (see discussion there).

The total cross section area has the same scaled coefficient of 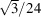 as in Equation 39 due to the same orientation of the NWire cross section (same growth vector) and thus the same small equilateral triangles as the unit area: 

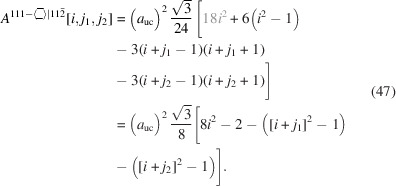

**Figure 7:**
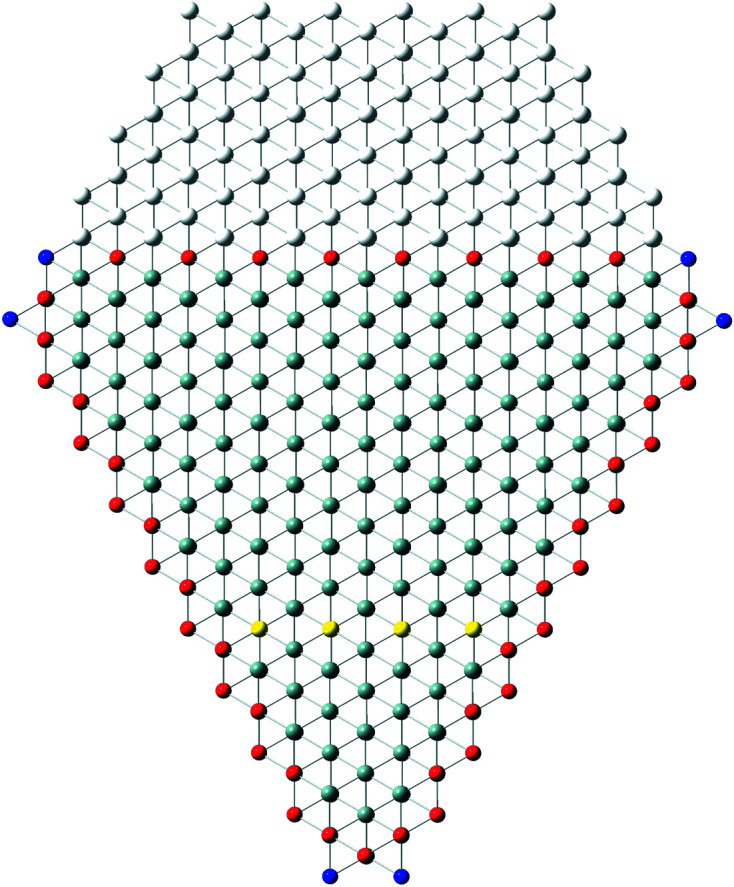
Axial-symmetric morphing of the zb-/diamond-structure NWires growing along the [111] axis with a hexa­gonal cross section and six {11 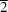 } inter­faces. The nominal cross section for *i* = 5 is shown by white ‘ghost atoms’ at the top inter­face and by the yellow atoms in the lower half of the cross section. Maximum reductive morphing with *j*_1_ = 4 was applied to the top inter­face, while morphing to maximum expansion with *j*_2_ = −(*i* −1) = −4 was applied to the bottom inter­face. For atom colours, refer to Fig. 4 ▸. For a detailed geometrical derivation of characteristic lengths and areas, refer to Appendix *C* [App appc].

The outermost area elements at the facets form isosceles triangles (Fig. 7 ▸), which have the same area as their equilateral counterparts mentioned above; see also the related discussion in Section 3.5 and the geometrical derivation explained in Appendix *C* [App appc]. The cross section of this NWire type is shown in Fig. 7 ▸.

## 4. Morphing cross sections along three symmetry axes

Such morphings naturally lend themselves to cross sections with hexagonal symmetry. We therefore do not consider square cross sections with 〈001〉 normal vectors on growth plane and facets, as well as rectangular cross sections with 〈110〉 growth vector and {001} and {110} facets.

Depending on the symmetry of the hexagonal cross section, we have to introduce different lateral number series per facet orientation, with run indices *j* as used in Section 3, and run indices 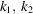 for the other two morphing directions, with a different facet orientation {*abc*} for both run indices 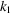 and 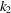 . This is the case in Sections 4.1 and 4.2.

**Figure 8:**
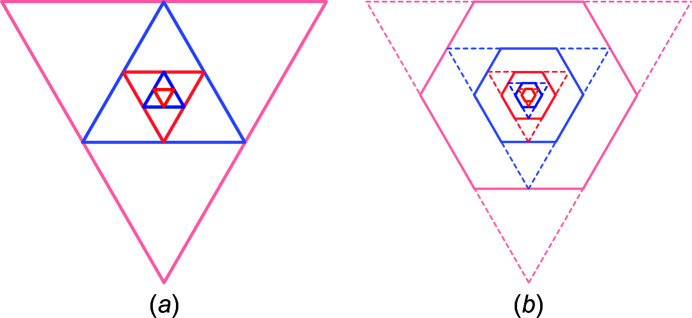
(*a*) Principle of exploiting the recursive (or fractal) symmetry properties of equilateral triangles to explain the coverage of arbitrary cross section shapes. Triangles represent the maximum expansive morphing for the respective nominal run index *i*. (*b*) Fitting the corresponding hexa­gon into these triangles shows the morphing range and size overlap, whereby the corresponding *i* values of cross sections shown differ by 2 or 3, depending on cross section type; see subsequent Figures in this section. Other cross sections between those shown – *i.e.* for all *i* values – have been omitted for clarity.

As in Section 3, lateral run indices – 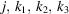 – are positive for reductive morphing (cutting into the nominal cross section) and negative for expansive morphing (extending the nominal cross section), with the nominal cross section presented if all lateral run indices are zero. Under the condition that all lateral run indices are equal, *i.e.* 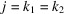 , or 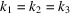 , all cross sections will assume a triangular or quasi-triangular shape on maximum expansive morphing, as well as on maxi­mum reductive morphing; see Fig. 8 ▸(*a*).

**Figure 9:**
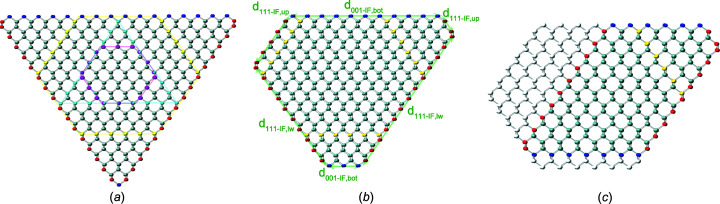
(*a*) Cross section of the regular hexa­gon with a [110] growth axis, {001} top and bottom inter­faces, and {111} side inter­faces, run index *i* = 6 and maximum extensive morphing (*j* = *k*_1_ = *k*_2_ = −5); regular cross section (*j* = *k*_1_ = *k*_2_ = 0) is shown by translucent yellow lines and nominal inter­faces in morphing directions are shown by yellow atoms. For atom colours, refer to Fig. 4 ▸. A smaller cross section [*i* = 3, *even* series; see König & Smith (2021 ▸)] is shown by purple atoms and the corresponding maximum expansive morphing by cyan atoms. Translucent lines were added to show the respective spatial limits. (*b*) Morphed cross section with *i* = 6, *j* = −3, *k*_1_ = −1 and *k*_2_ = −4. Inter­face lengths and associated labels are shown by translucent green lines, defining the area covered by the NWire cross section; see also Fig. 16 ▸ for outer triangular and offset sub-areas. (*c*) Morphed cross section with *i* = 6, *j* = 1, *k*_1_ = 4 and *k*_2_ = −4. White spheres present ‘ghost atoms’ to show nominal cross section. For a detailed geometrical derivation of characteristic lengths and areas, refer to Appendix *A* [App appa].

Other, more irregular, shapes can be described in an arbitrary fashion, under the constraint that all facets or singular points where facets meet do not penetrate the nominal hexagonal cross section. As a result, all facets not being directly morphed *via* a lateral run index have a minimum length or at least a point where adjacent (directly morphed) facets meet. These minimum lengths or singular points are all located on the respective borders (facets) of the nominal cross section considered. By preventing the penetration of such minimum facet lengths or singular points into the nominal cross section, we prevent the lateral number series from overlapping with each other, erasing the facet between the two associated morphing sections in the process. Thereby, we obtain a minimum facet length or a common point between two neighbouring morphed facets for maximum reductive morphing. A minimum facet length refers to cross sections morphed in Sections 4.1, 4.2 and 4.4, and a common point between two neighbouring facets refers to morphing in Section 4.3. While such overlap can be dealt with in number theory and crystallography, we point out that – besides its complexity – such a description of NWire cross sections is not beneficial since the free choice of the nominal run index *i* per cross section and subsequent morphing within these constraints covers virtually any convex NWire shape encountered in experiment. Apart from Section 4.3, where we introduce slightly different limits on run indices to prevent an overlap, such limitations are as follows. All lateral run indices have a minimum value of 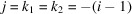 , resulting in maximum expansive morphing; see Figs. 9 ▸(*a*), 10 ▸(*a*) and 12(*a*). Run index doublets are limited to *j* + *k*_1_ = *j* + *k*_2_ = *k*_1_ + *k*_2_ = *i* − 1 in Sections 4.1 and 4.2, and to *k*_1_ + *k*_2_ = *k*_2_ + *k*_3_ = *k*_3_ + *k*_1_ = *i* − 1 in Section 4.4. The cross section treated in Section 4.3 has a limit on run index doublets of *k*_1_ + *k*_2_ = *k*_2_ + *k*_3_ = *k*_3_ + *k*_1_ = *i*, because its high symmetry and atom interconnectivity at the corner points allows for facets not being directly morphed to be reduced to singular points on the boundary of the regular cross section (*versus* minimum facet length for all other three cases). The basic principle of 3-axes morphing and related implications for overlap in size and form of cross sections is depicted in Fig. 8 ▸.

All number series reflect the variables we presented in Section 3, with additional series for facet lengths, NWire widths and heights, which depend on lateral (morphing) run indices. These are required in particular for finding the right indices to fit experimental values, such as facet lengths, heights or widths of NWire cross sections; see Section 5. As mentioned before in Section 3, the contribution of the nominal cross section to the respective number series is printed in grey to facilitate the decomposition into contributions per run index. For the same reason, most number series will be shown uncompacted, followed by their shortest form.

### 4.1. 3-Axes morphing of nanowires growing along the [110] direction and four {111} plus two {001} interfaces

For the number of atoms in the NWire cross section, we obtain 

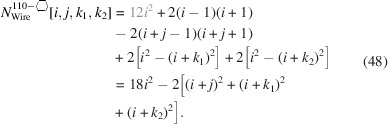

The number of bonds between these NWire atoms are described by 

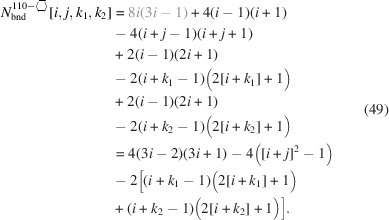

The total number of interface bonds of the NWire cross section amounts to 

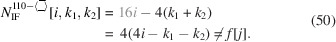

As was the case with axial-symmetric morphing (see Equation 16), the number of {111} facet bonds being added/removed equals the number of {001} facet bonds being removed/added per change in 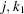 or 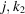 , whereby 
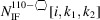
 becomes independent of *j*.

The ratio of facet bonds at {111} to {001} interfaces is given by 

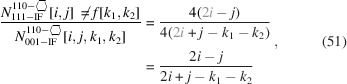

whereby the top row in Equation 51 show the explicit number series per interface orientation and the lower row presents their ratio. The length of the top 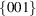 facet is given by 

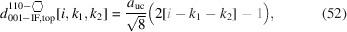

whereby the analogy of 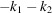 to 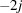 in clearly visible; see Equation 18. The length of the bottom {001} facet is given by 

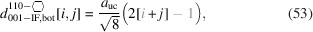

being equivalent to Equation 18.

The length of the two upper {111} facets depends only on the respective 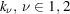 : 

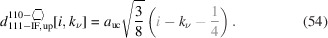

In Equation 54, we add or remove one *X*_6_ ring per change in 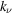 , as is the case for *j* in Equation 19, underlining the high symmetry of the NWire cross section. The length of the two lower {111} facets depends on the respective 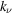 and *j*, the latter limiting such facets from below: 

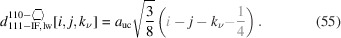

These facet lengths are shown in Fig. 9 ▸(*b*).

Due to 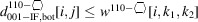 , the width of the cross section depends on 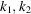 only, which is a direct consequence of the morphing limits discussed at the beginning of Section 4: 

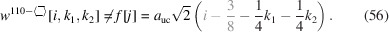

For the same reason, the height of the cross section depends only on *j*: 

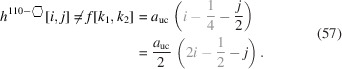

The total area of the cross section naturally depends on all running indices: 

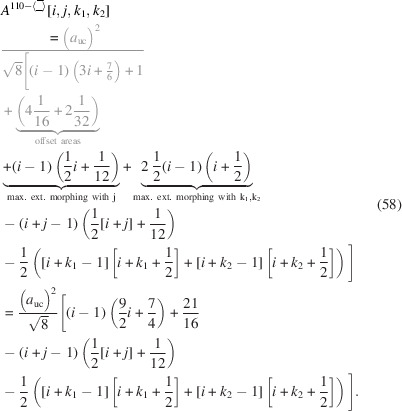

The underbrace in line 2 of Equation 58 indicates the offset area which is composed of four scalene and two isosceles triangles at the corner points of the cross section; see Appendix *A* [App appa] and Fig. 16 for their derivation^2^. The underbraces in line 3 of Equation 58 denote the contribution to maximum extensive morphing per class of lateral run indices *j* and 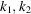 , from which the respective area is subtracted when 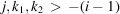 . Fig. 9 ▸ shows crystallographical details of this cross section and a couple of examples of triaxial morphing.

### 3-Axes morphing of nanowires growing along the [11 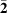 ] direction with four { 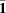 31} plus two {111} interfaces

4.2.

For the number of atoms in the NWire cross section, we obtain 

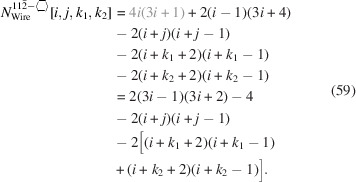

The number of bonds between these NWire atoms are described by 

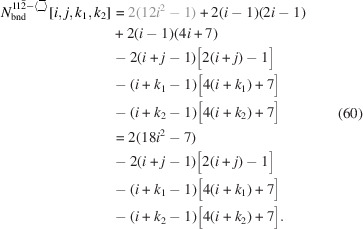

The total number of interface bonds of the NWire cross section amounts to 

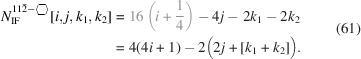

**Figure 10:**
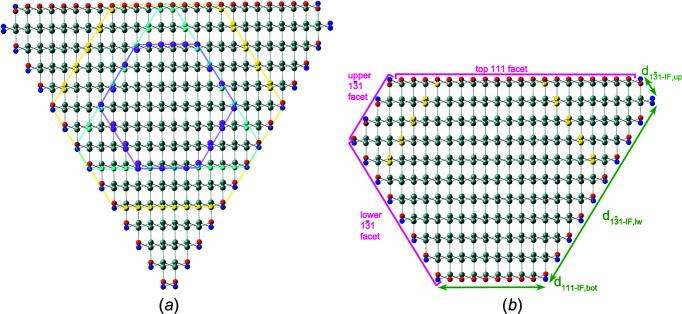
(*a*) Cross sections of regular hexa­gon with run index *i* = 5, a [11 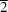 ] growth axis, {111} top and bottom inter­faces, and { 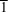 31} side inter­faces. The maximum expansive morphing shown corresponds to morph indices *j* = *k*_1_ = *k*_2_ = −4. For atom colours, refer to Fig. 4 ▸. A smaller cross section [*i* = 3, *even* series, see König & Smith (2021 ▸)] is shown by purple atoms and the corresponding maximum expansive morphing by cyan atoms. (*b*) Cross section with *i* = 5, *j* = 0, *k*_1_ = −2 and *k*_2_ = −4, with magenta lines assigning inter­face bonds to respective facets, and green arrows and labels showing inter­face lengths. For an example of reductive 3-axes morphing, refer to Fig. 2 ▸(*c*). For a detailed geometrical derivation of characteristic lengths and areas, refer to Appendix *B* [App appb].

The assignment of interface bonds to the {111} and {1 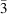 1} facets is shown in Fig. 10 ▸(*b*). With this assignment of interface atoms to {111} and {1 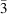 1} facets, we obtain 

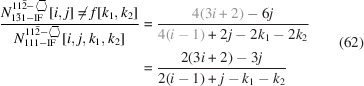

for the respective explicit number series in the top row and for the more compact form describing the ratio of interface bonds only. There are four different facet lengths which have to be used with their respective run indices *j* and 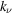 , 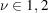 , as required for the facet of interest: 

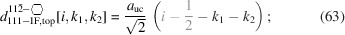




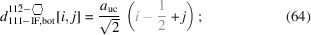




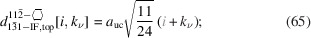




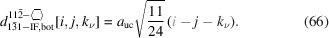

 For an illustration of facet lengths, we refer to Fig. 10 ▸.

As discussed in Section 4.1 around Equation 56, the width of the cross section depends on 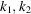 only: 

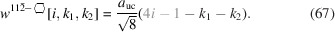

For the same reason, the height of the cross section depends only on *j*: 

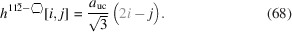

The total cross section area is presented by 

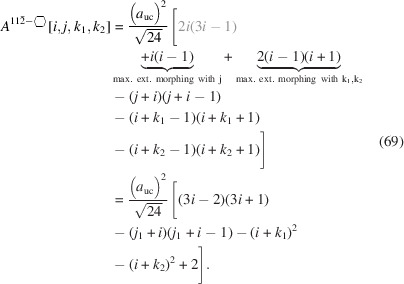

As for Equation 58, we have assigned the contribution to maximum extensive morphing per class of lateral run indices *j* and 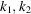 from which the respective area is subtracted when 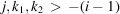 , before converting Equation 69 to its shortest form. For an illustration of irregular 3-axes morphing of this cross section, refer to Fig. 10 ▸.

### 4.3. 3-Axes morphing of nanowires growing along the [111] direction with a hexagonal cross section and six {110} interfaces

**Figure 11:**
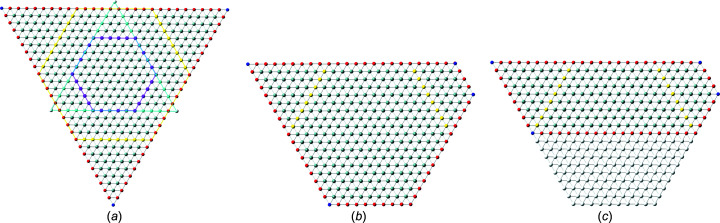
(*a*) Cross sections of a regular hexa­gon with run index *i* = 9, a [111] growth axis and six {110} inter­faces. The maximum expansive morphing shown corresponds to morph indices *k*_1_ = *k*_2_ = *k*_3_ = −9. For atom colours, refer to Fig. 4 ▸. A smaller cross section [*i* = 5, *even* series, see König & Smith (2021 ▸)] is shown by purple atoms and the corresponding maximum expansive morphing by cyan atoms. (*b*) Cross section showing expansive morphing, with *i* = 9, *k*_1_ = 0, *k*_2_ = −9 and *k*_3_ = −5. (*c*) Cross section showing reductive morphing, with *i* = 9, *k*_1_ = 9, *k*_2_ = −9 and *k*_3_ = −5. For a detailed geometrical derivation of characteristic lengths and areas, refer to Appendix *C* [App appc].

This cross section has a higher symmetry, as opposed to the two previous cases in Sections 4.1 and 4.2. All three morphing areas are identical and subject to their respective run index, which becomes apparent if we look at their interface orientations, which are identical to each other. As a result, we introduce just one class of run indices 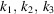 . We also point out that the morphing areas are identical to those depending on *j* in Section 3.5. The difference occurs by the morphing of opposite areas (referring to a *C*_2_ symmetry), while here we morph three equal areas – subject to identical run indices 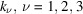 – when rotated by 120° (*C*_3_ symmetry). As there is no overlap in morphing regions for one 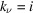 under the constraint that the other two *k* indices are ≤0 [see Fig. 11 ▸(*c*)], and the ultimate corner point of the extensive morphing occurs for 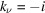 [see Fig. 11 ▸(*a*)], we can extend the 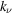 all the way to 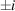 . Still, the indices 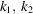 and 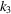 are restricted over 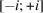 along 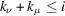 , where 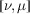 are cyclic permutations of the run indices, *viz.* 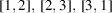 . Thereby, we avoid the morphing of the three triangular areas running into each other. As examples, if *i*  = 9 and 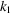 = 9, we have −*i* ≤ *k*_2_ + *k*_3_ ≤ 0; if *i*  = 9 and 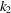 = 7, we have −*i* ≤ *k*_3_ + *k*_1_ ≤ 2, *etc*.

For the number of atoms in the NWire cross section, we obtain 

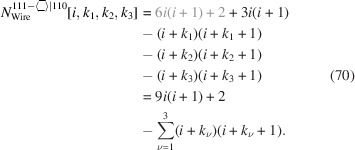

The final form of Equation 70 summarizes the terms which depend on 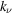 into a sum for brevity; we will use this short form in all subsequent equations where applicable. The number of bonds between these NWire atoms is described by 

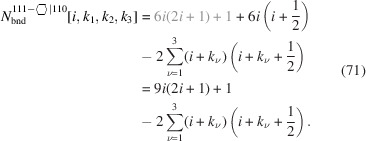

The total number of interface bonds of the NWire cross section amounts to 

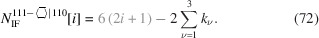

There are two types of interface lengths. One represents the facets normal to the morphing vector given by atomic planes being added or subtracted, and depends only on the respective 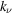 : 

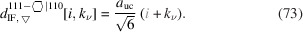

Another interface length exists for facets which are modified in their length by the two adjacent morphing regions: 

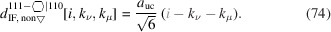

Due to the *C*_3_ symmetry of the hexagonal cross section, its height can be calculated along all three morphing vectors: 

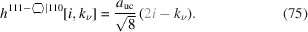

The calculation of the cross section width does not appear to be useful. It would require a discrimination to depart from the nominal width when 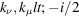 , adding an increment of 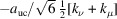 , which is somewhat cumbersome in handling experimental data. We therefore rely onto the height of the cross section as per Equation 75, which should be sufficient to assign run indices to an experimental image.

The total cross section area is presented by 

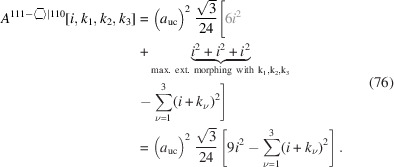

The maximum external morphing per 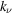 assigned in the top row of Equation 76 is straightforward to see in Fig. 11 ▸(*a*), where the three equilateral triangles cover half of the nominal cross section consisting of six of such triangles. Morphing examples are shown in Figs. 11 ▸(*b*) and 11 ▸(*c*).

### 3-Axes morphing of nanowires growing along the [111] direction with a hexagonal cross section and six {11 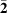 } interfaces

4.4.

This cross section reverts back to the restrictions on the run indices 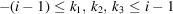 we had for cross sections in Sections 4.1 and 4.2, together with the restriction 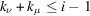 , where 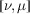 are cyclic permutations of the run indices; see beginning of Section 4. The symmetry considerations given in Section 4.3 also apply to this cross section which has exclusive { 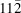 } facet orientations.

For the number of atoms in the NWire cross section, we obtain 

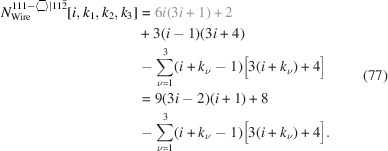

The number of bonds between these NWire atoms are described by 

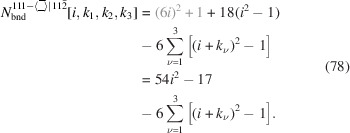

The total number of interface bonds of the NWire cross section amounts to 

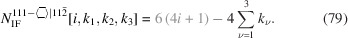

By analogy to Equations 73 and 74, we have two types of facet lengths with their respective lateral run indices, namely, the facets normal to the morphing vector given by atomic planes being added or subtracted 

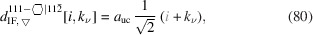

and for facets which are modified in their length by the two adjacent morphing regions: 

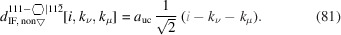

As was the case in Section 4.3, the *C*_3_ symmetry of the hexagonal cross section allows for its height to be calculated along all three morphing vectors: 

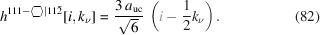

The total cross section area is presented by 

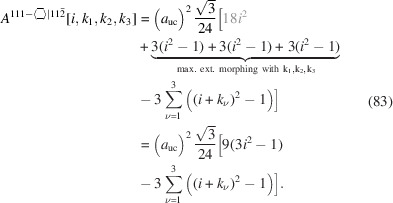

**Figure 12:**
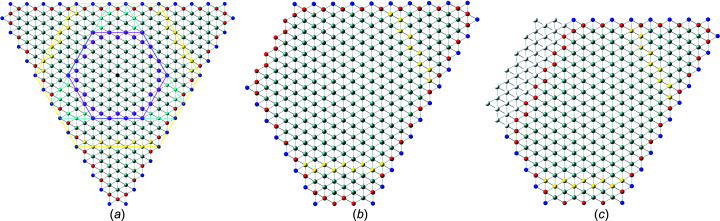
(*a*) Cross sections of a regular hexa­gon with run index *i* = 5, a [111] growth axis and six {11 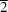 } inter­faces. The maximum expansive morphing shown corresponds to morph indices *k*_1_ = *k*_2_ = *k*_3_ = −4. The two black atoms are located in the centre of the cross section in the same lateral position, *i.e.* on top of each other. For other atom colours, we refer to Fig. 4 ▸. A smaller cross section [*i* = 3, *even* series, see König & Smith (2021 ▸)] is shown by magenta atoms and the corresponding maximum expansive morphing by cyan atoms. (*b*) Cross section showing expansive morphing, with *i* = 5, *k*_1_ = −2, *k*_2_ = 0 and *k*_3_ = −4. (*c*) Cross section showing reductive morphing, with *i* = 5, *k*_1_ = −1, *k*_2_ = 2 and *k*_3_ = −4. For a detailed geometrical derivation of characteristic lengths and areas, refer to Appendix *C* [App appc].

The maximum external morphing per 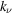 assigned in the top row of Equation 83 is straightforward to see in Fig. 12 ▸(*a*), where the three axial-symmetric trapezoids fold back onto the nominal cross section, which consists of six such trapezoids, whereby the two central atoms shown in black in Fig. 12 ▸(*a*) are not covered in the folding process. Morphing examples are shown in Figs. 12 ▸(*b*) and 12 ▸(*c*).

## 5. Application examples

### 5.1. Si NWires

**Figure 13:**
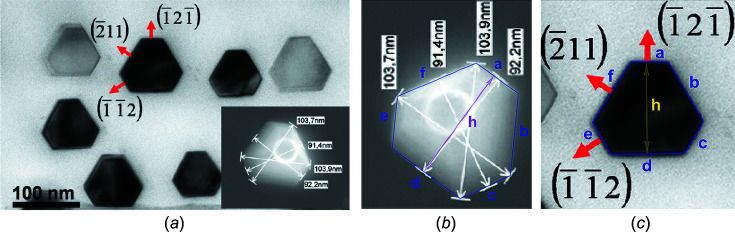
Cross sections of Si NWires growing along the [111] axis and {110} inter­faces from Moutanabbir *et al.* (2011 ▸). (*a*) Reprinted with permission from Moutanabbir *et al.* (2011 ▸), copyright American Chemical Society 2011. Examples of (*a*) for gauging with number series are shown enlarged in parts (*b*) and (*c*); see Table 4 ▸ for the parameters of the respective NWire cross section.

Si NWires have been shown to grow as monolithic crystals along the [111] axis with atomically flat 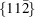 interfaces when aluminium (Al) is used as a seed catalyst (Moutanabbir *et al.*, 2011 ▸). Such NWire cross sections are shown in Fig. 13 ▸. We picked two examples from this reference to show the usage and results derived by the morphing algorithms from experimental input. Table 4 ▸ shows all parameters and results of the cross sections shown in Figs. 13 ▸(*b*) and 13 ▸(*c*), respectively.

Due to several run indices present, the fitting onto the exact cross section shape requires an iterative process which is well suited to a computer code. Such a code could be added to existing visual software for gauging NWire cross sections – a task we illustrate here in a stepwise fashion as a principal guide. As unit-cell parameter for Si, we use 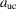 = 0.54309 nm (Böer, 1990 ▸).

There are two ways to start an iteration for obtaining the run indices. The first is to start with one 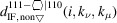 (Equation 74) and its two adjacent 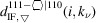 (Equation 73), rearranging for the three run indices such as 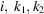 . This approach may be more appealing to the experimentalist, and is illustrated on a core-shell NWire in Section 5.2. The more direct starting point for the iteration is given by using 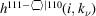 (Equation 75) and the 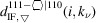 , which is at one end of 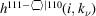 , rearranging only for the two run indices involved.

With the measured height and interface lengths *h* and *a*, as listed for Fig. 13 ▸(*b*) in Table 4 ▸, we get 

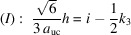




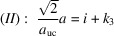




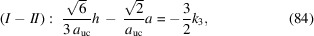

yielding 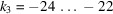 and *i* = 127. The tolerance range for 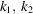 and 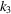 originates from the tolerance in length measurement which is in the range 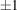 nm; see Fig. 13 ▸ and Table 4 ▸. Such tolerance ranges translate into ranges for 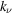 and *i* which serve to minimize the difference to the respective calculated length 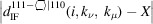 ; see Equation 85 below and Equation 86 in Section 5.2. With *i* known, we can proceed with Equation 84(*II*) to get a start range for the other two 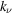 , yielding *k*_2_(*i* = 127, c) = 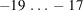 and *k*_1_(*i* = 127, e) = 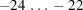 . Next, we rearrange Equation 74 for its run indices, *viz.*


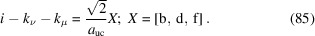

Equation 85 provides us with a sum of 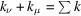 , which we can match under consideration of the range of 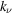 and 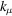 . For 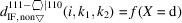 , we get 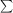 *k* = −43 … −41 = *k*_1_ + *k*_2_. This range allows for 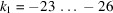 and *k*_2_ = −17 … −19. Moving on to 
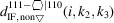
 = *f*(*X* = b), we obtain 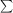 *k* = −43 … −41 = *k*_2_ + *k*_3_. This range allows for 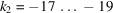 and 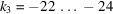 , with the valid doublets of 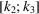 = [−17;−24], [−18;−23], [−19;−22], [−18;−24], [−19;−23] and [−19;−24]. Arriving at the last interface length 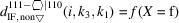 , we get 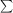 *k* = −45 … −43 = *k*_3_ + *k*_1_, allowing for 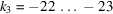 and *k*_1_ = −22 … −23, yielding 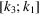 = [−23;−22], [−22;−23], [−22;−22]. It becomes apparent that with ongoing iteration over an increasing number of interface lengths 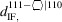 , the 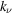 indices get narrowed down towards one integer value. We can narrow down the range for the 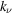 further by reconsidering Equation 84(*I*) with *i* = 127 and 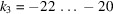 to match the experimental value *h* [Fig. 13 ▸(*b*) and Table 4 ▸]. This is best achieved using 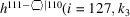 = −22) = 91.8 nm, leaving just 0.4 nm to match the measured value of *h*. With 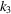 = −22, we can iterate again, obtaining 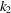 = −19 and 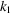 = −23 ± 1  = −23, whereby we chose the centre of the 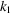 range to arrive at a minimum deviation from the measured length parameters of the NWire cross section. An iterative computer code would modify 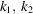 and 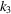 around their initial values such that the sum of the absolute deviation values of all the NWire length parameters from their measured counterparts 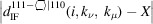 is minimized as the criterion to arrive at the best structural fit of the number series; see Equation 86 in Section 5.2. The indices [*i*, *k*_1_, *k*_2_, *k*_3_] = [127, −23, −19, −22] can then be used in Equations 77 to 83 to calculate the structural results. These are shown in Table 4 ▸, together with the results for the cross section in Fig. 13 ▸(*c*).

The flexibility of the above algorithms in describing the NWire cross section could also be very useful for NWire shapes changing by post-growth extrinsic means. As an example, an atomic rearrangement at Si NWires due to high current densities inducing local heating (Bahrami *et al.*, 2021 ▸) can be tracked and linked to the surface energies of respective facets. Tracking such changes over time with our crystallographic description and the energy intake by local heating should allow the atomic surface diffusion process to be described in much detail. Such findings can be key to NWire design on demand.

### 5.2. Core-shell III–V zb-NWires with different unit-cell parameters *a*_uc_

**Figure 14:**
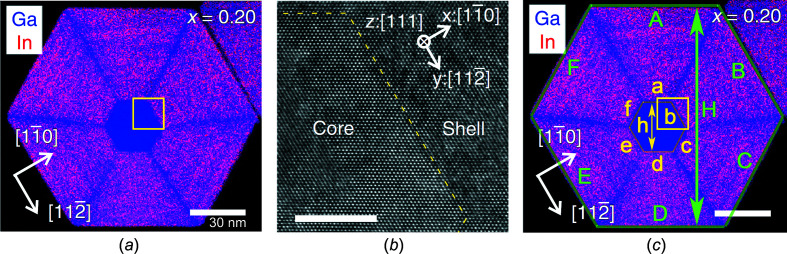
Core-shell GaAs–In_0.2_Ga_0.8_As zb-NWire with a [111] growth axis and {110} inter­faces [Balaghi *et al.* (2019 ▸); reproduced with kind permission of Springer Nature, copyright 2019]. (*a*) Original elemental distribution in NWire cross section obtained by energy-dispersive X-ray spectroscopy. (*b*) Transmission electron micropscopy image of the NWire region around the inter­nal inter­face between GaAs and In_0.2_Ga_0.8_As, as shown by the yellow square in part (*a*). The inter­face is monocrystalline and assumed to be atomically flat. (*c*) Core-shell NWire cross section with inter­faces illustrated and labelled for crystallographic analysis as per number series in Section 4.4.

III–V NWires are often found to grow along the [111] axis which requires the least energy and have hexagonal cross sections (Joyce *et al.*, 2011 ▸; Treu *et al.*, 2015 ▸). Here we will focus on core-shell GaAs–In_0.2_Ga_0.8_As zb-NWires grown by solid-state molecular beam epitaxy (MBE) along the [111] axis with {110} interfaces, using visual and crystallographic data from Balaghi *et al.* (2019 ▸). Fig. 14 ▸ shows the NWire cross section with crystallographic details and the assignment of variables to respective interface lengths and one UC height of the NWire cross section.

Below, we will show how we can derive structural results using Equations 70 to 76 in Section 4.3 to match all interface lengths to their measured value (Table 5 ▸), taking the different unit-cell parameters for core and shell of NWire, 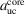 and 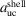 , into account. The resulting run indices allow us to obtain the main variables 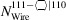 , 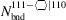 , 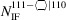 and 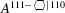 for the core and total NWire cross sections, hereafter denoted as 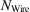 , 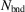 , 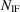 and *A*, respectively, to keep the prsentation of vari­ables as simple as possible. The same simplification goes for all 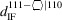 and 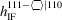 , hereafter denoted as 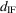 and *h*, respectively. From these data, we can derive the main variables of the shell by simple differential/additive calculations. The calculation of run indices for the core and shell of the NWire cross section require additional indexing of the run indices to avoid confusion: run indices using the unit-cell parameter of the NWire core 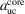 will be 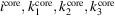 , and run indices using the unit-cell parameter of the NWire shell 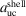 will be 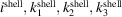 .

We set out with the cross section of the NWire core (Fig. 14 ▸) and use the lengths of three adjacent interfaces as a starting point, thereby illustrating the second method briefly men­tion­ed in Section 5.1 of how to find the run indices 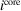 , 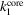 , 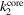 , 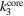 NWire cross sections.

The convergence criterion (*CC*) we use for obtaining the run indices which describe the NWire cross section with minimum deviation is given by the sum of absolute deviations of the calculated 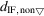 , 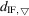 and 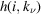 – see Equations 73 to 75 in Section 4.3 – from their respective measured values a to f plus *h*_1_ to *h*_3_ for the NWire core, and A to F plus *H*_1_ to *H*_3_ for the NWire shell; see Fig. 14 ▸(*c*): 

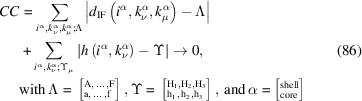

Please observe that below we will work with only one height per cross section to keep the explanation of the fitting procedure concise and Fig. 14 ▸(*c*) readable; the inclusion of all three heights into a computer code featuring Equation 86 is straightforward. As a starting point for all cross sections, we set 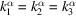 = 0 (presenting the nominal regular shape of the NWire cross section) and run an iteration scheme with Equation 86 in compound with Equation 87, using *i* ^α^ as a run index to arrive at an educated guess from where to start the fitting procedure.

Our search criterion features a length of an interface which depends on three run indices, 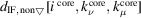 , plus its adjacent interface lengths 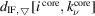 and 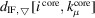 . We start with choosing 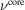 = 1 and 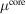 = 2 to obtain the following convergencies: 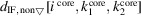 → d, 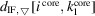 → e and 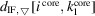 → c, with c, d and e being the measured interface lengths listed in Table 5 ▸: 

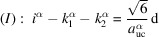




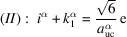




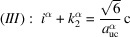




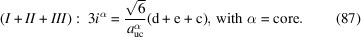

When treating the cross section of the NWire shell, we have to use the interface lengths presented by capital letters in Fig. 14 ▸(*c*) – c, d and e change to C, D and E – and set α = shell in Equation 87. The last line of Equation system 87 adds up Equations (*I*) to (*III*), thereby eliminating all 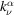 , yielding the search condition for a regular hexagonal cross section to make an educated guess at a starting value for 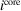 . The unit-cell parameter we use is the value for GaAs, 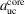 = 0.56533 nm (Böer, 1990 ▸), which comprises the NWire core; see Fig. 14 ▸. If *i* ^α^ is located nearly halfway between two integer values, we may run the calculations below with both adjacent *i* ^α^ values to see which one has the lower *CC* as per Equation 86. Once we got 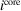 , we can run each single Equation 87 (*I*) to (*III*) for obtaining 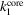 and 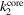 . We then move on to the next 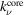 and 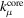 , namely, 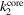 and 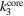 , and to the next three measured interface lengths e, f and a. The third index rotation then uses 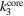 and 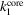 with the interface lengths a, b and c. As a result, we obtain the final results for the NWire core, 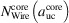 , 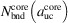 , 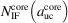 and 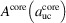 . When iterating for the NWire shell cross section, we proceed the same way, using the adequate variables as mentioned above.

As in Section 5.1, we use the tolerance in length measurement (Fig. 14 ▸ and Table 5 ▸) to provide narrow ranges for all *k* ^α^ and *h* ^α^ around their calculated value, aiming for a minimum *CC* while keeping *i* ^α^ constant. For the NWire core, the result of this fitting procedure with *CC* → min are the run indices 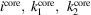 and 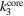 . Replacing a to e and h_1_ to h_3_ in the above process with A to E and H_1_ to H_3_, and using 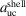 = 0.57343 nm (Adachi, 2004 ▸) as the unit-cell parameter of the shell material featuring In_0.2_Ga_0.8_As, we get 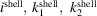 and 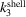 for the NWire shell. All these indices are shown in Table 5 ▸, together with the measured interface length and height of the respective cross section [Fig. 14 ▸(*c*)].

The run indices of the NWire core cross section can be used directly to calculate all of its parameters discussed in Section 4.3. Deriving the parameters of the shell and eventually of the entire NWire system requires some additional calculations. After calculating 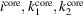 and 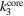 with 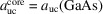 , we apply an increment to 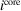 , *viz.* 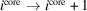 , and use the same 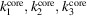 for a calculation of 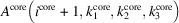 with 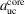 . We then calculate the cross sections of the core with 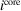 and 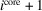 and the same 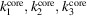 as obtained with 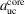 . The transition 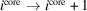 adds one atomic ML to the cross section, accounting for the interface region between the core and shell material as seen from the NWire core using 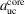 : 

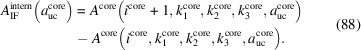

We then iterate again as per the above description for the NWire core, but now use 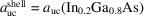 , see the third row in Table 5 ▸, obtaining 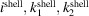 and 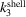 .

With this result, we again apply the increment 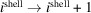 and use the same 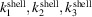 for a calculation of 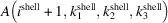 with 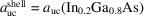 . This transition adds one atomic ML to the core cross section as well, using the unit-cell parameter of the shell material, accounting for the interface region between the core and shell material, as seen from the NWire shell using 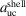 : 

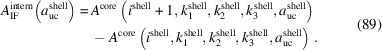

With both interface areas, we can now calculate their average value as the most accurate interface area we can obtain: 

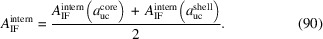

The reason we use two descriptions of the interface area is given by the transition of the unit-cell parameters when going from the core to the shell material. This approach can be further exploited for an ML-wise increment in cross section with 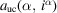 for each increment in *i* ^α^, which adds further precision if a radial distribution of the unit-cell parameter is known, such as in Fig. 3(*a*) in Balaghi *et al.* (2019 ▸), and is further discussed in Section 5.2.1. The indices 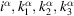 and 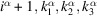 of the NWire core cross section without and with one ML as interface region for each, 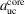 and 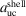 , have further use for other interim data we use to arrive at our final results. Before we can derive 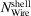 , 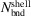 , 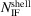 and 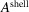 of the NWire shell, and 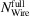 , 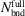 , 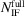 and *A* ^full^ of the complete NWire, we need to carry out another iteration scheme using 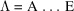 and 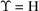 (Equation 86) for the total NWire cross section with 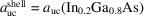 . The resulting 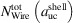 , 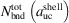 and 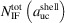 with their respective running indices are then used in straightforward differential calculations.

The calculation of the number of atoms within the NWire shell is obtained from 

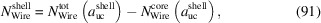

thereby eliminating all atoms from the total NWire area in the core region. The calculation of 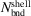 requires 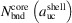 , where we use 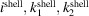 and 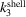 obtained in Equation 89. In addition, we have to substract 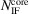 , as such bonds belong to the interface region between the core and shell: 

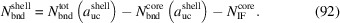

From a practical viewpoint, the calculation of 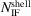 delivers two values. For spectroscopic characterization techniques where no carrier recombination is involved, such as Raman, Fourier-transform infrared (FT–IR) or electron paramagnetic resonance, the core-shell interface bonds 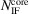 are considered as dipoles whereby they get counted only once with the core for the complete NWire, resulting in 
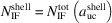
 . For spectroscopic characterization techniques where carrier recombination is involved, such as photoluminescence or carrier lifetime spectroscopy, the interface bonds at the core-shell interface can acquire and trap free carriers from the core, as well as from the shell. Hence, these bonds have an impact on the core *and* shell, whereby we count 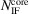 for 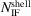 in addition to including them for the core: 

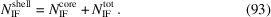

Since the number of interface bonds does not directly depend on 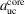 *versus* 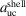 for epitaxial NWire growth, we can drop their dependence on the unit-cell parameter in Equation 93. The area of the NWire shell requires the area of the NWire core and the core-shell interface region to be subtracted from the total NWire area, *viz.*


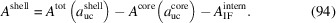

The final results of the full NWire cross section – core and shell with their respective *a*_uc_ – follow from the addition of results from core and shell, namely, 

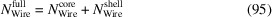




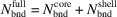




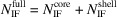




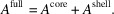

The area of the internal interface 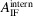 (Equation 90) is another final result not included in *A* ^full^, since NWire interfaces behave in a significantly different manner from the core and shell regions in terms of electronic and optical properties, such as carrier transport, recombination and interface dipoles. The calculation of the dimensionless crystallographic parameters 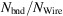 , 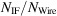 and 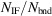 , which allow for inter-NWire comparison, are straightforward. Table 5 ▸ shows all the measured lengths of the core-shell NWire depicted in Fig. 14 ▸, together with selected interim results for the cross section of the NWire core using 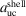 listed in column 3, and all final results.

#### 5.2.1. Flexibility of cross section calculations for core-shell NWires

We have briefly mentioned above that – due to *i* → *i*+1 adding a defined ML (usually atomic ML) to the NWire cross section – we can introduce a unit-cell parameter with a radial dependence 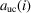 to the entire core-shell NWire. If such a dependence is known, *e.g.* for the NWire shell (Balaghi *et al.*, 2019 ▸), the precision of the NWire description can be further increased. We note here that non-radial deviations of 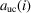 , such as local inhomogeneities, cannot be accounted for due to the radial layer dependence of all number series with their main run index *i*.

**Figure 15:**
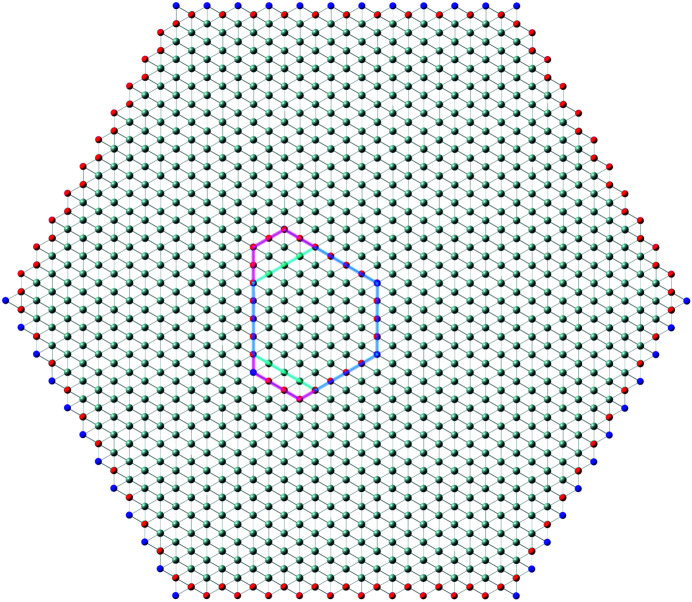
Cross section of the core-shell NWire growing along a [111] axis. The cross section of the NWire core has {110} inter­faces, is irregular (*i* ^core^ = 4, *k*_1_^core^ = −2, *k*_2_^core^ = 0 and *k*_3_^core^ = −1) and located off-centre with respect to the symmetry centre of the NWire shell. The NWire shell has {11 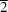 } inter­faces and is regular (*i* ^shell^ = 12, *k*_1_^shell^ = *k*_2_^shell^ = *k*_3_^shell^ = 0). Arbitrary cross section shapes as per individual morphing can be combined if the core and shell share the same symmetry (crystal orientation) along the growth axis. The circumference of the NWire core is highlighted by a magenta line and the corresponding regular NWire core is shown by a cyan line.

**Figure 16:**
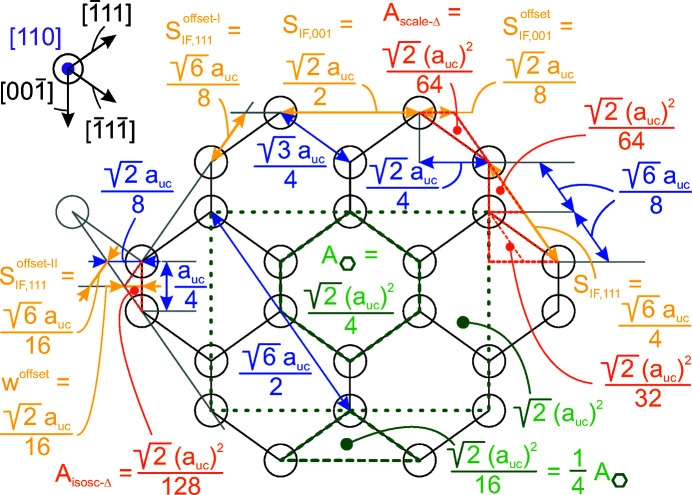
Geometric relations for the zb-structure along the [110] growth vector. The UC in the {110} plane is shown by a dotted dark-green line. The unit area 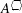 (grey–green) for the {110} plane is defined by the area of a six-membered ring. The distance increments required for calculating the lengths of the {100} and {110} inter­faces are shown by *s*_IF,111_ and *s*_IF,100_, respectively (dark yellow), together with their respective offsets *s* 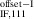 , *s* 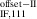 and *s* 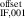 . The offset in NWire width *w* ^offset^ is shown in dark yellow as well. All offset areas are shown in orange, namely, one of the four scalene triangles 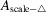 , and one of the two isosceles triangles 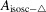 . All other length parameters are shown in blue. A scheme of the relevant lattice vectors within the {110} plane is shown on the upper left.

Since our analytic treatment of zb-NWire cross sections works on the basis of smallest area segments coming along with every atom and bond considered, the arrangement of the core and shell to each other is flexible over a wide range. To illustrate the implications, the NWire core does not have to be located in the centre of the NWire shell, nor does any restriction exist for the core and shell NWire cross sections to be morphed independently from each other. This finding can be verified in our above example (Fig. 14 ▸ and Table 5 ▸). It becomes apparent from Fig. 14 ▸ that the NWire core is not aligned with the NWire shell to share the same symmetry centre. We can go further down this path and adapt the outer NWire shape to a different interface orientation, as would be the case for a core-shell NWire growing along the [111] axis, with internal {110} and external 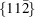 interfaces (Fig. 15 ▸). Such an NWire cross section requires a partition into three sections, of which two are treated in accord with the core and shell sections in the above numerical example (Fig. 14 ▸). The third section describes the outermost shape of the NWire shell, where the change in interface orientation occurs. When assembling the respective variable 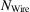 , 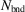 , 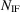 and *A* for the entire NWire, we calculate the full shell (index ‘tot’ in above example) of the entire NWire with outer 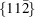 interfaces and then simply substract the core cross section with 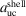 , yielding the above variables for the shell with different faceting at the inner and outer interfaces. Such core-shell NWire descriptions can be chained to describe multi-core-shell cross sections by repeating the calculations shown in this section for every core-shell pair.

## 6. Conclusions

Building on our previous work (König & Smith, 2021 ▸), we introduced extensions into analytical number series for zb- and diamond-structure NWires for adapting their cross sections to arbitrary shape (morphing), covering the following NWire cross sections: square, 〈001〉 growth axis and interfaces; rectangular, 〈110〉 growth axis and {110} plus {001} interfaces; hexagonal, 〈110〉 growth axis and {001} plus {111} interfaces; hexagonal, 〈 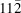 〉 growth axis and {111} plus 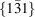 interfaces; hexagonal, 〈111〉 growth axis and {110} interfaces; hexagonal, 〈111〉 growth axis and 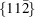 interfaces. Our extensions provide the exact crystallographic description of zb-NWires with arbitrary cross sections as encountered in experiment, and thus are only limited in their precision by measurement tolerances of the imaging technique used. As previously, the results we obtain by our analytics are the number of NWire atoms 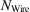 , the number of bonds between such atoms 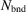 , the number of NWire interface bonds 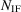 and cross section areas *A*. We demonstrated that our analytic description is applicable with the same precision to core-shell NWires with arbitrary shape and interface orientation of the core and shell, under the constraint that they share the same orientation of their growth axis, and have an interfaces roughness below the tolerance limit of the measured interface lengths. The above results are available per core and shell section of the NWire, and internal (core-shell) interface areas are given as well. If a radial distribution of the unit-cell parameter can be provided, such data can be included for all mentioned NWire cross sections, adding further flexibility and precision to their description. The description of core-shell NWires can easily be applied to multiple core-shell (layered) NWires if these comply with epitaxial growth and smooth interfaces.

The analytic description of zb- and diamond-structure NWire cross sections with arbitrarily convex shape and multiple radial layers (multiple core-shell structures) can provide major advancements in experimental data interpretation and understanding of III–V, II–VI and group-IV-based NWires. The number series allow for a deconvolution of experimental data into environment-exerted, interface-related and NWire-internal phenomena. Our method offers an essential tool to predict NWire cross sections and to tune process conditions for tailoring NWires towards desired shape and interface properties, see König & Smith (2019 ▸), König & Smith (2021 ▸) and König (2016 ▸) for details.

## Figures and Tables

**Figure 17:**
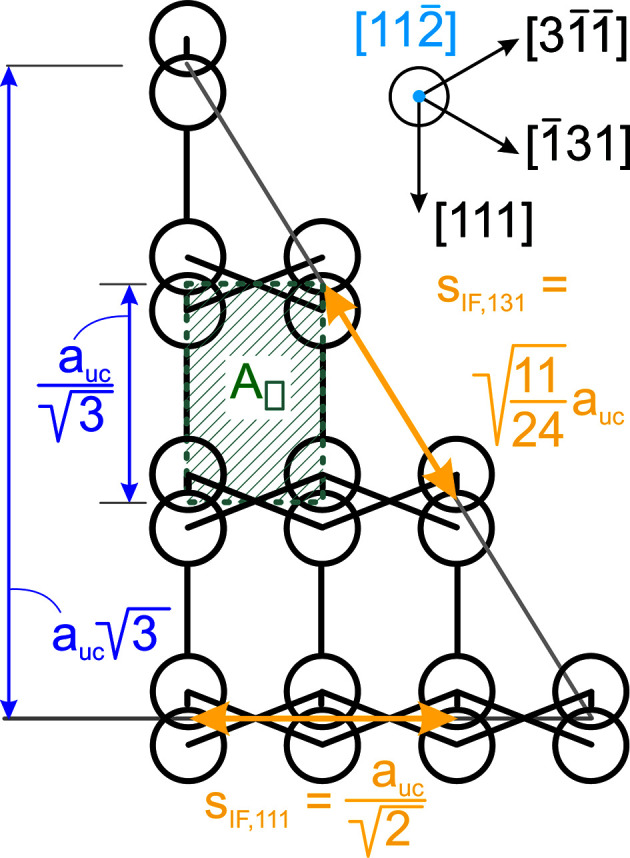
Geometric relations for the zb-structure along the [11 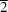 ] growth vector. The rectangle enclosed by the dotted dark-green line presents the unit area *A*_ 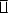
_ of the {11 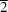 } plane. The distance increments required for calculating the lengths of the {100} and {110} inter­faces are shown by *s*_IF,131_ and *s*_IF,111_, respectively. A scheme of relevant lattice vectors within the {11 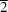 } plane is shown on the upper right.

**Figure 18:**
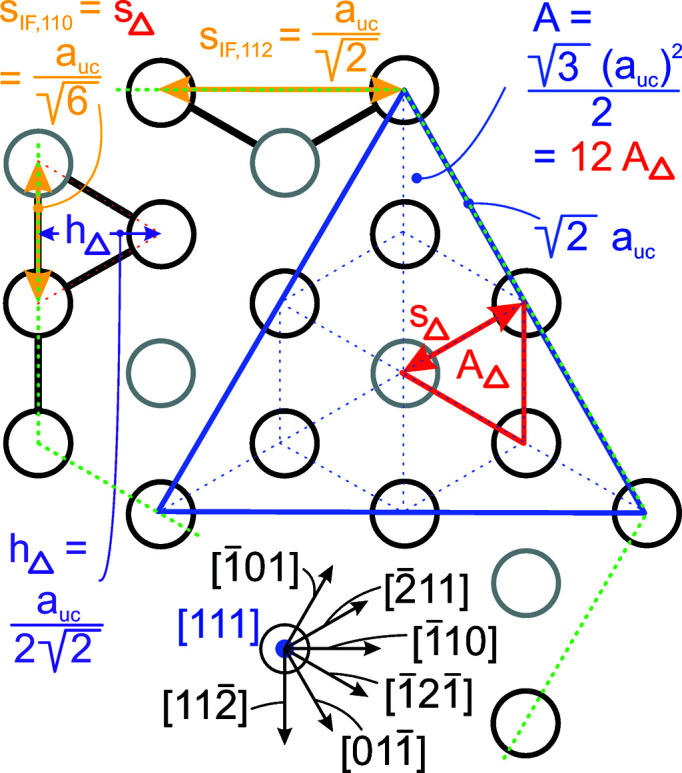
Geometric relations for the zb-structure along the [111] growth vector. The cross section through the UC in the {111} plane is shown by the blue triangle. The unit area *A*_Δ_ for the {111} plane is shown in red. The distance increments required for calculating the lengths of the {100} and {110} inter­faces are shown by *s*_IF,110_ and *s*_IF,112_, respectively (dark-yellow lines). Dotted green lines mimic the {111} and {11 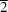 } inter­faces. A scheme of the relevant lattice vectors within the {111} plane is shown at the bottom.

**Table 1:** Primary (top) and secondary (bottom) variables calculated by the analytic description of zb-NWire cross sections as per König & Smith (2021 ▸)

Formula sign (unit)	Parameter description
*N_Wire_	Number of NWire atoms
*N_bnd_	Number of inter­nal NWire bonds
*N_IF_	Number of NWire inter­face bonds
*d*_IF_ (nm)	Inter­face length
*N_*abc*-IF_	Number of NWire bonds, inter­face {*abc*}
*d*_*abc*-IF_ (nm)	Inter­face length, orientation {*abc*}
*A* (nm^2^)	Cross section area
*N*_bnd_/*N*_Wire_	Bonds per atom within zb-NWire
*N*_IF_/*N*_bnd_	Inter­face bonds per inter­nal NWire bond
*N*_IF_/*N*_Wire_	Inter­face bonds per NWire atom
*N*_*abc*-IF_/*N*_*def*-IF_	Inter­face bond ratio between facet

Note: (*) numbers refer to an NWire slab of 1 UC thickness along the growth vector; see König & Smith (2021 ▸) for details.

**Table 2:** Slab thickness *d*_slab_ of NWire cross sections as a function of the growth axis orientation given in UC lengths *a*_uc_ per growth orientation to achieve periodicity. Numbers of atoms and of bonds per column as described per feature seen in cross section top view are given to enable the counting of atoms and NWire-inter­nal bonds

	*d*_slab_	Atoms	Bonds
Growth axis	[*a*_uc_]	Per column, in top view	
001	1	1	1 per / and \
110	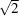	2	2 per / and \, 4 per |
111	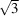	2	1 per atom column, 1 between atom columns
11 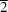	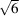	1	2 per 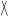 , 1 per — and –†

Note: (†) bond symbols must be turned by 90° to align with the graphs in Figs. 2 ▸ and 10 ▸.

**Table 3:** List of NWire shape indices – cross section, growth direction and side inter­faces (where necessary) – added to all parameters as a superscript

Superscript	Growth axis	Nominal cross section	Side inter­faces†
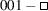	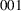	Square	
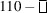	110	Rectangular	
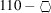	110	Hexa­gon	
11 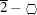	11 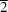	Hexa­gon	
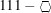 |110	111	Hexa­gon	110
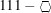 |11 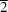	111	Hexa­gon	11 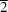

Note: (†) only when required to distinguish cross sections.

**Table 4:** Structural parameters and calculated results for hexa­gonal cross sections with a [111] growth axis and {11 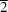 } inter­faces, as shown in Figs. 13(*b*) and 13(*c*). The slab thickness of NWire cross sections is *d*_slab_ = *a*_uc_ 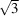 . Measured lengths are shown with tolerances

Measured parameter (nm)	See Fig. 13(*b*)	See Fig. 13(*c*)
a	40.8 ± 1.0	33.2 ± 1.0
b	64.1 ± 1.0	59.2 ± 1.0
c	41.9 ± 1.0	33.4 ± 1.0
d	64.9 ± 1.0	60.0 ± 1.0
e	39.8 ± 1.0	32.1 ± 1.0
f	65.8 ± 1.0	60.5 ± 1.0
h	92.2 ± 1.0	79.8 ± 1.0
		
Calculated parameter		
*i*	127	109
*k*_1_	−23	−25
*k*_2_	−19	−22
*k*_3_	−22	−23
*N*_Wire_/*d*_slab_	335796	255450
*N*_bnd_/*d*_slab_	669937	509449
*N*_IF_/*d*_slab_	3310	2902
*A* ^ 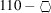 ^ (nm^2^)	7130	5422
*N*_bnd_/*N*_Wire_	1.995	1.994
*N*_IF_/*N*_Wire_	9.857 × 10^−3^	1.136 × 10^−2^
*N*_IF_/*N*_bnd_	4.941 × 10^−3^	5.696 × 10^−3^

**Table 5:** Parameters for hexa­gonal core-shell NWire cross section as per Fig. 14(*c*). The slab thickness of NWire cross sections is *d*_slab_ = *a*_uc_ 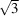 . Data for the In_0.2_Ga_0.8_As cross section of the NWire core are listed in column 3 where necessary to explain calculated parameters. Measured lengths are shown with tolerances. See text for more details

Material	GaAs	In_0.2_Ga_0.8_As	In_0.2_Ga_0.8_As	
*a*_uc_ (nm)	0.56533	0.57343	←	
				
Measured parameter (nm) 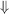	NWire core (a–f, h)	NWire core (a–f, h)	NWire shell (A–F, H)	Full NWire
a, A	15.2 ± 0.4	←	68.0 ± 0.8	←
b, B	15.2 ± 0.4	←	69.0 ± 0.8	←
c, C	14.4 ± 0.4	←	66.2 ± 0.8	←
d, D	15.9 ± 0.4	←	69.4 ± 0.8	←
e, E	14.9 ± 0.4	←	68.2 ± 0.8	←
f, F	15.5 ± 0.4	←	69.0 ± 0.8	←
h, H	26.2 ± 0.4	←	117.3 ± 0.8	←
				
Calculated parameter				
*i*	66	65	290	
*k*_1_	−1	1	−1	
*k*_2_	−2	2	6	
*k*_3_	1	0	−1	
*N*_Wire_/*d*_slab_	26794	25344	485608	512402
*N*_bnd_/*d*_slab_	53187	50298	969053	1022240
*N*_IF_/*d*_slab_	802		3502/4304*	4304/5106*
*A* (nm^2^)	608.8		10861	11470†
*A* 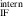 (nm^2^)				18.5
*N*_bnd_/*N*_Wire_	1.985		1.9955	1.9950
*N*_IF_/*N*_Wire_ (×10^−3^)	29.93		7.212/8.863*	8.863/9.965*
*N*_IF_/*N*_bnd_ (×10^−3^)	15.08		3.614/4.441*	4.210/4.995*
*A* ^core^/*A* ^full^				0.0531†

Notes: (*) bonds of the inter­nal inter­face can contribute once/twice to a specific property considered, hence are counted once/twice; see explanation in the text. (†) Excludes area of core-shell inter­face *A* 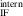 ; see explanation in the text.

## Footnotes

1 We also note here that the offset area was corrected again with respect to König & Smith (2021 ▸). This correction amounts to a mere 0.0644  , which translates to *ca*. 1.95 Å^2^ for semiconductors such as Si or GaAs, and hence should have no practical relevance in experiment.

2 We also note here that the offset area was corrected again with respect to König & Smith (2021 ▸). This correction amounts to a mere 0.0644  which translates to *ca*. 1.95 Å^2^ for semiconductors such as Si or GaAs, and hence should have no practical relevance in experiment.

## Appendix A Geometric details for NWire cross sections with [110] growth vector

We start the analysis with the zb-UC projection into the {110} plane which defines the NWire cross sections; see Fig. 16 ▸. We then look at unit lengths to calculate the interface lengths, width and height of the cross section. For {001} facets, this unit length is  . For the {111} facets, its unit length  is straightforward to obtain from the 〈  〉 diagonal through the {110} plane of the UC, which is  . Amounting to half of the length of this diagonal, we get  . Each facet has length offsets which are required to arrive at the correct interface length, width and height of the cross section. For  , two identical offsets exist, each being  . The total offset is thus  ; see Equations 52 and 53. For the {111} facets, two offset lengths  and  exist.

For deriving  , we use the  diagonal through the {110} plane of the UC. When going through the {110} plane along the  vector, we see that we have four equi­distant atomic MLs. These divide the  diagonal of the UC into four equal parts which each fit  . We therefore get  . For deriving  and the width offset  , we use  and the inter-ML distance in the  direction, which is  . We first note that we can use the intercept theorem to obtain  , hence  , and thus  . *Via* the opposite corner, the total width offset is then  , adding 1/8 to the −4/8 offset in Equation 56 3. With  obtained, we can calulate  using Pythagoras’ theorem, *viz.*

The total offset for the {111} facet lengths is thus  . Taking  into account, we get  . Noting that  , we see the offset  implemented in Equations 54 and 55 by the substraction of 1/4 from the run index *i*. The offset in *h* becomes apparent when looking at the number of atomic MLs increasing in the  direction with *i* as 3, 7, 11, … for *i* = 1, 2, 3, *etc*. The −1 ML offset to  thus accounts for the offset of −1/4 as per Equation 57.

Next, we work out the areas which form the total cross section. The hexagonal rings in the {110} plane are the basic area unit of this cross section, amounting to  =  =  , which is equivalent to 1/4 of the {110} plane of the UC. Breaking down  further, we arrive at the area for the open isosceles triangles at the {001} facets which each have an area of  . These two areas are required to calculate the cross section area as a function of run indices. There are six offset areas presented by one triangle at each corner of the hexagon which can be calculated by using the offset lengths derived in the previous paragraph. Two isosceles triangles exist at the two corners between {111} facets. Their area is straightforward to derive from Fig. 16 ▸ as  =  =  . Each of the four corners between the {001} and {111} facets has a scalene triangle as an offset area. We can use translational, reflectory and rotational geometry operations, as illustrated in Fig. 16 ▸, to show that  =  . Adding up all offset areas, we get

With the prefactor of the unit area  used in Equation 58, we finally get

## Appendix B Geometric details for NWire cross sections with [11 ] growth vector

In contrast to the rather non-trivial  growth vector class, the geometrical details of the  plane class cross section are rather simple, mostly owing to the  top and bottom facets. We start with the diagonal through the  plane of the UC, as shown in Fig. 17 ▸, amounting to  . This length is composed of three bonds which lie in the  plane, with each being  long, plus three bonds sticking out of the plane at an angle of  = 54.73° with a projected length in the  plane of  each. From these two lengths, we get the bigger length of the unit area  as  =  , or alternatively *via*  =  . This length presents the increment unit for the height of the cross section; see Equation 68. The unit length of the  facet along the  vectors is given by  (see Equations 63 and 64). With these two lengths, we can calculate the unit area required to obtain the total area of the cross section as  =  =  (see Equation 69), whereby we used  =  . The length  is also used as the unit length for the width of the cross section; see Equation 67. The diagonal of this rectangular unit area presents the unit length of the  facets as  ; see Equations 65 and 66.

## Appendix C Geometric details for NWire cross sections with [111] growth vector

We start our analysis by deriving the unit area, followed by the derivation of unit lengths for the width, height and facets per cross section type.

The unit area used for calculating the total area of NWire cross sections with [111] growth vector  is given by the equilateral triangle shown in red in Fig. 18 ▸. Its area is straightforward to obtain by projecting the {111} plane cut through the UC into the scheme as shown by the blue large triangle in Fig. 18 ▸. This equilateral triangle has a side length of  , which is twice as big as the unit length for the  facets;  . The {111} plane cut through the UC has a total area of  and consists of six equilateral triangles plus six isosceles triangles. All of these triangles have the same area, as is apparent from the thin dotted blue lines within the {111} plane of the UC in Fig. 18 ▸ with the use of some trivial mirror-symmetry operations. We thus get the unit area of the cross section as  , see the prefactor in Equations 76 and 83. The side length  of the unit area  follows straight from the fact that this triangle is equilateral as well, resulting in  . Hexagonal symmetry shows that  is also the unit length of the {110} facets;  , see the prefactor in Equations 73 and 74, and 1/3 of the increment in height for cross sections with  facets, *cf.* the prefactor in Equation 82. Looking at  from the perspective of the  facets, we get the increment in height for cross sections with  facets as *h*_Δ_ =  ; see the prefactor in Equation 75. This Equation makes it clear that the transition  for  is required for cross section morphing to accommodate height changes with  .
