# Analytic description of nanowires II: morphing of regular cross sections for zincblende- and diamond-structures to match arbitrary shapes. Corrigendum

**DOI:** 10.1107/S2052520623000434

**Published:** 2023-03-14

**Authors:** Dirk König, Sean C. Smith

**Affiliations:** a Integrated Materials Design Lab (IMDL), Research School of Physics and Engineering, The Australian National University, ACT 2601, Australia; b Institute of Semiconductor Electronics (IHT), RWTH Aachen University, 52074 Aachen, Germany; c Integrated Materials Design Centre (IMDC), University of New South Wales, NSW 2052, Australia; d Department of Applied Mathematics, Research School of Physics and Engineering, The Australian National University, ACT 2601, Australia

**Keywords:** nanowire cross section, metrology, morphing, zincblende, diamond

## Abstract

Corrections to the article by König and Smith [*Acta Cryst.* (2022), B**78**, 643–664 ▸] are given.

## Abstract 2

Corrections to the article by König and Smith [ *Acta Cryst.* (2022), B**78**, 643–664] are given.

In the paper by König & Smith [*Acta Cryst*. (2022), B**78**, 643–664 ▸], a number of printing errors occurred. Two equations suffer from a sign error at a lateral running index, namely Equations 18 and 54. In §5.1, several typographical errors occurred when referring to equation numbers, and in the superscript indices of lengths *h* and interface lengths *d*_IF_. In addition, the caption of Fig. 13 wrongly mentions {110} instead of 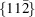 interfaces, and the caption of Fig. 14 refers to §4.4 instead of §4.3. Such errors do not alter any analytical, numerical or other findings of the paper. We provide all corrections in tabular form. Apart from Equations 18 (§3.3) and 54 (§4.1), and the caption of Fig. 14, the misprints are all located in the text of §5.1 and its accompanying Fig. 13, ranging from the beginning of the third paragraph (‘There are two ways ...’) to the end of Equation (85). Table 1 ▸ lists the original strings which were misprinted and their correct version in the sequence as they appear in the original paper.

The authors apologize for inconveniences caused by the misprints.

## Figures and Tables

**Table 1:** Corrections to text

Version in published paper	Correct version
Section 3.3	
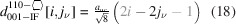	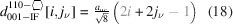
Section 4.1	
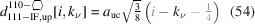	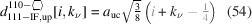
Section 5.1	
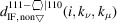 (Equation 74)	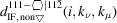 (Equation 81)
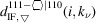 (Equation 73)	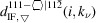 (Equation 80)
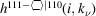 (Equation 75)	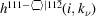 (Equation 82)
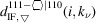	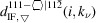
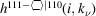	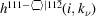
Fig. 13, caption: 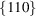 interfaces	Fig. 13, caption: 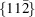 interfaces
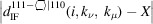	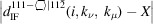
Equation 74	Equation 81
Section 5.2	
Fig. 14, caption: in Section 4.4	Fig. 14, caption: in Section 4.3
